# High-Pressure Polymorphism of Ribavirin

**DOI:** 10.1021/acs.cgd.5c00372

**Published:** 2025-05-12

**Authors:** Bhaskar Tiwari, Hanns-Peter Liermann, Simon Parsons, Nico Giordano

**Affiliations:** † Centre for Science at Extreme Conditions and School of Chemistry, 3124 The University of Edinburgh EH9 3FJ Edinburgh, U.K.; ‡ 28332 Deutsches Elektronen-Synchrotron DESY, Notkestr. 85, 22607 Hamburg, Germany

## Abstract

The effect of high pressure on ribavirin, a broad-spectrum antiviral consisting of ribofuranosyl triazole, and carboxamide moieties, has been studied up to ∼10 GPa. Three new high-pressure phases, designated **V3**, **V4** and **V5**, have been obtained by compression of the ambient-pressure **V2** form with structures refined up to 7.5 GPa. The new phases are formed at 5.3, 6.0, and 7.2 GPa, respectively, and crystallize in space group *P*2_1_2_1_2_1_ with *Z*′ = 3, 1, and 1. They are distinguished by changes in the conformation of the ribofuranosyl moiety which impacts both the molecular geometry and the supramolecular structure.

## Graphical abstract

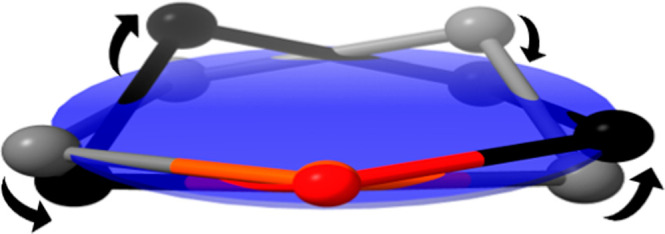

## 1 Introduction

Exhaustive characterization of solid forms of active pharmaceutical ingredients (APIs) is essential in drug discovery, as different solid forms can vary significantly in their physicochemical properties,^1^ potentially leading to problems during manufacturing, storage, and drug delivery. Solid form screening and selection are therefore vital stages of the drug discovery pipeline.^2^

Traditional methods for screening a wide range of polymorphs, solvates, hydrates, salts, and cocrystals focus on modifying crystallization conditions, such as varying solvents/antisolvent mixtures, or temperature and drying conditions at ambient pressure.^2^ In addition, high-pressure techniques are increasingly employed to explore a broader range of phase-space, providing additional insights into the solid-state behavior and stabilities of APIs. Applied pressures range from a few hundred MPa, which simulate pressures typically encountered during manufacturing or tableting processes, up to several GPa, where crystal packing, molecular conformations, and intermolecular interactions are readily altered.^3,4^ These studies may consist of compression of known ambient pressure solid forms, recrystallization of solutions at high pressure to overcome kinetic barriers, or solid-to-solid transformations, to access novel polymorphs, solvates, or cocrystals.^3,5^

High-pressure phase transitions in molecular solids are fundamentally linked to the reconfiguration of intermolecular interactions driven by the minimization of volume. Alternative arrangements of intermolecular interactions are often close enough in energy that a small penalty in lattice energy is compensated for by the pressure × volume contribution to the overall free energy of a new phase. This concept extends to conformational changes in flexible molecules,^6^ particularly in ring systems such as the furanose units of nucleosides which are often used as antiviral APIs.^7^ The conformations of five membered furanosyl rings have been the subject of a number of detailed structure correlation studies which have demonstrated that conformational shifts are characterized by small energy differences between twisted and envelope forms around a pseudorotation pathway.^8,9^ This conformational flexibility is an important feature in nucleosides, impacting their role in nucleic acid structures, enzyme recognition, and pharmaceutical applications.^10^

The structure and dynamics of nucleosides have been probed by spectroscopic techniques in between 1 and 6 GPa. In crystalline adenosine, changes in vibrational signatures of the furanosyl ring at 2.5 GPa suggest a modification in ring puckering corresponding to a pressure-induced phase transition.^11^ Similar transitions have been suggested in thymidine and cytidine at 3 and 4 GPa.^12^ Structural studies by single-crystal diffraction are less common, and while the furanosyl ring features in one study of sucrose to 6 GPa, glucose and two forms of mannose at high pressure have reported changes in pyranosyl rings.^13^ A search for high-pressure structures containing nucleosides in the Cambridge Structural Database (CSD) v5.45 (November 2024) results in no hits. Among macromolecules, Girard et al.^14^ have reported a small modification of the sugar puckering parameter in the d­(GGTATACC) oligonucleotide at ∼1 GPa by synchrotron single crystal X-ray diffraction.

The structural effects of pressure on furanosyl ring conformations above 0.1 GPa thus remain largely unexplored. This study seeks to address this gap by examining how conformational flexibility influences the phase behavior of the API and nucleoside analog ribavirin, while also exploring the structural effects of pressure and the potential of high-pressure techniques to uncover novel solid forms, as demonstrated for other APIs.^15^ Ribavirin (1-(β-d-ribofuranosyl)-1,2,4-triazole-3-carboxamide) is a small-molecule, broad-spectrum antiviral agent used in the treatment of various viral infections, including respiratory syncytial virus and hepatitis C.^16^ It has two known polymorphs under ambient conditions, named **V1** and **V2**, which differ both conformationally and in terms of their packing.^17,18^ While **V1** is the denser form, **V2** is the thermodynamically stable form due to stronger cohesive energies within the crystal.^19^

## 2 Experimental Section

### 2.1 Crystallization and Preparation

Ribavirin was obtained from Sigma-Aldrich (98%) and the **V2** form was recrystallized from methanol by evaporation at room temperature. In a typical experiment, the needle-like crystals were cut using a razor blade (50 μm × 25 μm × 30 μm) and loaded in a short-symmetric piston cylinder type diamond anvil cell (DAC) offering an 80° opening, along with a ruby sphere. The DAC was equipped with type Ia Boehler-Almax diamonds with culet size of 500 μm and a preindented rhenium gasket of thickness between 60–85 μm, with a spark-eroded sample chamber with an initial hole diameter of 250 μm. Six separate compression studies were carried out using neon or nitrogen as a pressure transmitting medium (PTM). In each case, the identity of the **V2** polymorph was verified prior to introduction of the PTM by single crystal X-ray diffraction at ambient conditions. Loading of the medium was accomplished using a Sanchez Technologies gas loading system. The pressure inside the cell was determined using the ruby fluorescence method; under hydrostatic conditions, the precision of pressures measured by ruby fluorescence is typically 0.05 GPa.^20^

### 2.2 Single Crystal X-ray Diffraction (SC-XRD)

SC-XRD data were collected at ambient pressure on a Bruker D8-Venture diffractometer (Mo Kα radiation, λ = 0.7107 Å). High-pressure diffraction data were collected up to 10 GPa at the Extreme Conditions Beamline P02.2 at PETRA III (DESY, Hamburg, Germany) using an X-ray energy of ∼43 keV (λ ≈ 0.2900 Å) with a focused beam size of horizontal and vertical dimensions ∼2 × 2 μm^2^ or ∼8 × 3 μm^2^. Data were measured across a φ-rotation range of ±35° with steps of 0.5° and an acquisition time of 1 s per image using a PerkinElmer XRD1621 detector. Data completeness, which is always restricted at high pressure by the opening angle of the DAC, was in the range 56 to 65%.

### 2.3 Data Processing

Apex4 and CrysAlisPRO were used for data integration and reduction.^21^ Corrections for systematic errors such as absorption and, in the case of the high-pressure data, gasket shadowing, were carried out using the multiscan method.^22^ Structures were solved using dual-space methods in SHELXT and SHELXD as implemented in Olex2, and refined against |*F*|^2^ with SHELXL.^23^ The coordinates of the C, N and O atoms were refined freely, but the limited completeness required the use of enhanced rigid-bond restraints to stabilize the refinement of the anisotropic displacement parameters.^24^ H atoms were placed on carbon and nitrogen in idealized positions. H atoms attached to oxygen in the ambient conditions structure were initially refined as freely rotating rigid groups to find the most favorable conformation, and thereafter allowed to ride on their parent atoms. Subsequent structures in the parent phase were then refined with these fixed hydrogen positions due to decreased data quality at high pressure. The hydrogen positions of the hydroxyl groups in the **V3** system were refined freely. Difference maps calculated for **V4** at 6.0 and 7.0 GPa appeared to indicate different orientations of the hydroxyl group based on O3. The treatment of these hydrogens will be discussed in Section 3.5. Selected structure and refinement data are listed in Table 1; a full set is available in Table S1 in the Supporting Information. Crystal structures have been deposited in the CSD under deposition numbers CCDC 2392034–2392063.

**Table 1:** A Summary of Selected Crystal Structure and Refinement Data

pressure (GPa)	0.00	0.91	2.38	5.49	5.35	5.96	7.18
phase	V2	V2	V2	V2	V3	V4	V5
crystal data							
a, b, c (Å)	5.2848(5)	5.2243(1)	5.12795(16)	5.0082(3)	5.0234(2)	4.9465(3)	5.1260(3)
	7.7035(8)	7.5753(10)	7.4600(9)	7.3176(10)	21.287(3)	6.2449(10)	5.970(2)
	24.982(2)	24.4047(12)	23.7653(15)	23.030(3)	23.590(2)	25.978(2)	25.389(3)
V (Å^3^)	1017.05(17)	965.83(14)	909.13(13)	843.99(16)	2522.6(5)	802.46(16)	777.0(3)
Z	4	4	4	4	12	4	4
data collection							
no. of measured, independent and observed [I > 2σ(I)] reflections	14,610	2414	1875	1993	5307	1999	1451
	2083	1082	1038	1010	2847	951	749
	1318	1032	980	966	2330	863	650
*R*_int_	0.087	0.020	0.029	0.023	0.048	0.038	0.031
refinement							
*R*[F^2^ > 2σ(F^2^)], wR(F^2^), S	0.053	0.048	0.031	0.030	0.044	0.041	0.045
	0.153	0.233	0.088	0.083	0.100	0.111	0.124
	1.02	1.13	1.17	1.12	1.00	1.15	1.05

Three new high-pressure polymorphs of ribavirin have been identified in this study and these will be referred to as **V3**, **V4**, and **V5** to be consistent with the phase nomenclature established in the literature.^17^ The quality of the data obtained permitted structure refinements up to 7.5 GPa. Beyond this pressure only cell dimensions are reported.

### 2.4 Other Programs Used

Structure visualization and root-mean-square deviation (RMSD) calculations of atomic positions were conducted in Mercury.^25^ Mogul was used for statistical assessment of internal geometric parameters.^26^ Third order Birch–Murnaghan equations of state (EoS) were used to fit pressure–volume data (EoSFIT7-GUI).^27^ The underlying topology of the crystal structures was analyzed using ToposPro.^28^ Ring puckering analysis using the Altona model was carried out using PLATON.^29^ The molecular volume for **V2** was calculated using MolVol which calculates the volume of a molecule based on atomic van der Waals radii using a Monte Carlo algorithm, while the void volume was calculated using the CellVol program.^30^ Intermolecular energies were calculated using the semiempirical PIXEL method, implemented using the MrPIXEL program in Mercury.^31^ Electron density calculations were carried out using Gaussian09 at the MP2 level of theory with a 6-31G** basis set.^32^

Geometry optimizations of the alternative structures for both **V4** structures were calculated using the DMol3 program using periodic density functional theory (DFT) as implemented in the Materials Studio suite.^33^ The Perdew–Burke–Ernzerhof (PBE) functional was used as the generalized gradient approximation (GGA) to determine the most energetically favorable hydrogen positions in the **V4** structures. Dispersion was treated with the Tkatchenko–Scheffler method and the unit cell dimensions were held fixed at their experimental values.^34^

## 3 Results and Discussion

### 3.1 Ambient Pressure Structure

**V2** crystallizes in space group *P*2_1_2_1_2_1_ with four molecules in the unit cell, and one molecule in the asymmetric unit The atomic numbering scheme (Figure 1) for non-H atoms follows the previously published entry in the CSD (reference code VIRAZL01).^35^

**Figure 1:**
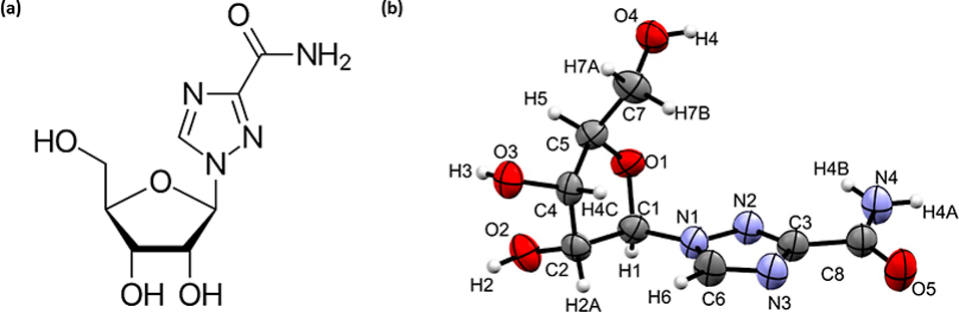
(a) Structural formula of ribavirin (b) molecular structure of the **V2** form at ambient pressure showing the atomic numbering scheme. Ellipsoids are shown at the 50% probability level.

Ribavirin consists of a 1, 2, 4-triazole-3-carboxamide moiety connected to a ribofuranose (ribose) sugar unit via a glycosidic bond. While the bond distances do not lie beyond normal ranges, two angles, both formed at the ribose-substituted planar N1-atom of the triazole ring, lie slightly above and below the usual ranges for chemically similar fragments: C1–N1–C6 [134.0(6)°, MOGUL^26^ *Z*-score 2.330] and C6–N1–N2 [108.9(4)°, *Z*-score 2.039]. Two torsion angles along the glycosidic bond also have very small local density scores (LSD) retrieved from the CSD for such structures: C2–C1–N1–N2 [−179.5(4)°, LSD 0.016] and C2–C1–N1–C6 [2.0(8)°, LSD 0.007].^36^ Five-membered rings can be described by envelope (E) and twist (T) conformations, where one or two atoms lie outside of the mean ring plane, respectively[Fn fn1]· ^9,37^ Puckering analysis of the ribose unit indicates that the furanosyl ring adopts an E_2_ conformation with the C2 atom sitting outside the plane of the rest of the ring, with a pseudorotation phase angle, *P*, of 336.0(4)°.

The first coordination sphere of **V2** at ambient pressure consists of six pairs of symmetry-related molecules, creating a topologically cubic close packed structure (Figure 2).^38^ The lattice energy of **V2** at ambient pressure, as calculated with the Pixel method, is −227.8 kJ mol^–1^. The energies of the principal intermolecular interactions are shown in Table 2 where the interactions are ordered by energy and labeled in symmetry-related pairs from *A*/*A*′ to *F*/*F*′.

**Figure 2:**
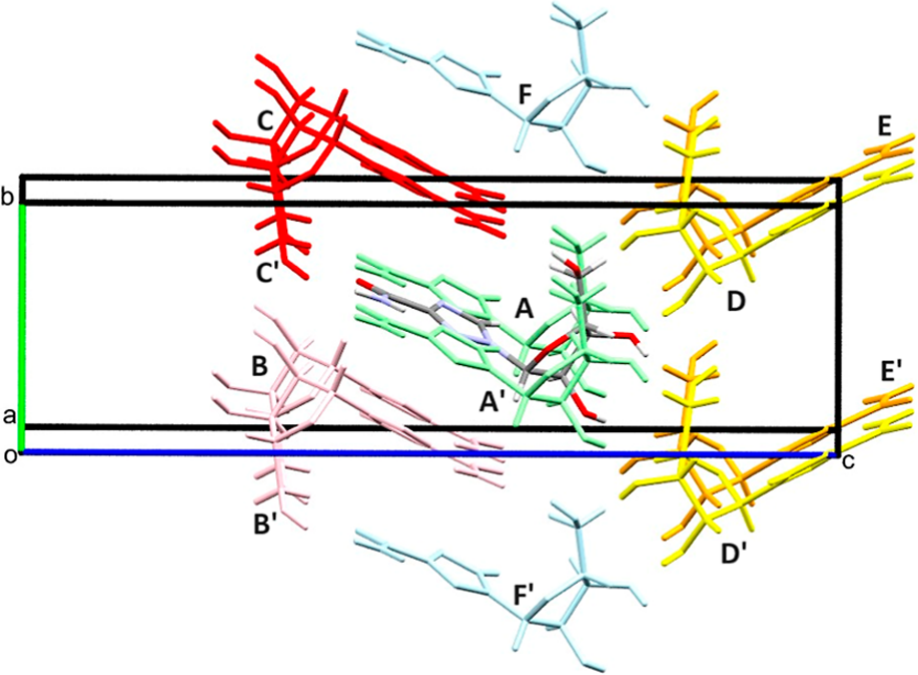
First coordination sphere of the **V2** form at ambient pressure viewed down the *a*-axis. The 12 molecules surrounding the central reference molecule form 6 symmetry equivalent pairs. Symmetry equivalent molecules are denoted with an apostrophe.

**Table 2:** Intermolecular Energies Present in the First Coordination Sphere of Ribavirin at Ambient Pressure, and the Breakdown of These Energies Portraying the Strength of Different Energetic Contributions^a^

symmetry transformations	interaction label	centroid distance (Å)	coulombic	polarization	dispersion	repulsion	total	contacts
*x* + 1, *y*, *z*	*A*/*A*′	5.285	–79.8	–31.6	–46.8	82.3	–75.9	2xN4H4B···N3 = 2.09 Å, ∠ = 164.2°
*x* – 1, *y*, *z*								
*x* – 1/2, –*y* + 1/2, –*z* + 1	*B*/*B*′	6.780	–21.4	–7.3	–30.5	19.6	–39.7	dispersion dominant interaction
*x* + 1/2, –*y* + 1/2, –*z* + 1								
*x* – 1/2, –*y* + 3/2, –*z* + 1	*C*/*C*′	7.713	–56.6	–30.1	–25.4	73.7	–38.5	2xO4H4···O5 = 1.87 Å, ∠ = 168.0°
*x* + 1/2, –*y* + 3/2, –*z* + 1								
–*x* + 1, *y* – 1/2, –*z* + 3/2	*D*/*D*′	8.270	–61.4	–25.5	–21.5	73.0	–35.5	2xO3H3···O4 = 1.90 Å, ∠ = 161.6°
–*x* + 1, *y* + 1/2, –*z* + 3/2								
–*x* + 2, *y* – 1/2, –*z* + 3/2	*E*/*E*′	8.590	–39.9	–18.6	–16.4	51.2	–23.6	2xO2H2···O3 = 1.96 Å, ∠ = 157.2°
–*x* + 2, *y* + 1/2, –*z* + 3/2								
*x*, *y* + 1, *z*	*F*/*F*′	7.703	2.6	–0.8	–5.7	1.5	–2.4	nonspecific long–range interaction
*x*, *y* – 1, *z*								

a The energies are given in kJ mol^–1^, and the negative sign denotes a stabilizing interaction. H-bonds are defined as described by Wood et al.^39^

The strongest H-bonded interaction (*A*) involves Coulombic attraction (Figure 3) via lattice translations along **a** with the molecules linked by N4H4B···N3 H-bonds between amide and triazole groups (molecule–molecule interaction energy = −75.9 kJ mol^–1^, N4H4B···N3 = 2.09 Å, ∠N4H4B···N3 = 164.2°). The interaction also includes a short C6H6···O1 contact (H6···O1 = 2.36 Å, ∠C6H6···O1 = 174.2°) involving the triazole ring and the oxygen atom in the neighboring furanose ring. Interaction *C* (−38.5 kJ mol^–1^) contains an O4H4···O5 H-bond connecting the hydroxymethyl group of the furanosyl ring and the carbonyl oxygen of the carboxamide group (O4H4···O5 = 1.87 Å, ∠O4H4···O5 = 168.0°). Interactions *A* and *C* generate a double-layered infinite chain which is approximately rectangular in cross section with oxygen-based donor and acceptor groups distributed on the short sides of the rectangle (Figure 3). These motifs are obliquely stacked along **b**. Formation of the stacks does not involve any conventional H-bonding contacts, and the molecules instead interact through interaction *B* (−39.7 kJ mol^–1^), a mixed electrostatic–dispersion interaction with alignment of positively and negatively charged regions along the long molecular axes. The stacking also features the weakly stabilizing interaction *F* (−2.4 kJ mol^–1^). The stacks are connected into a herringbone like arrangement through OH···O contacts between hydroxyl groups: *D* (O3H3···O4 = −35.5 kJ mol^–1^, 1.90 Å, ∠O3H3···O4 = 161.6°) and *E* (O2H2···O3 = −23.6 kJ mol^–1^, 1.96 Å, ∠O2H2···O3 = 157.2°), as shown in Figure 3.

**Figure 3:**
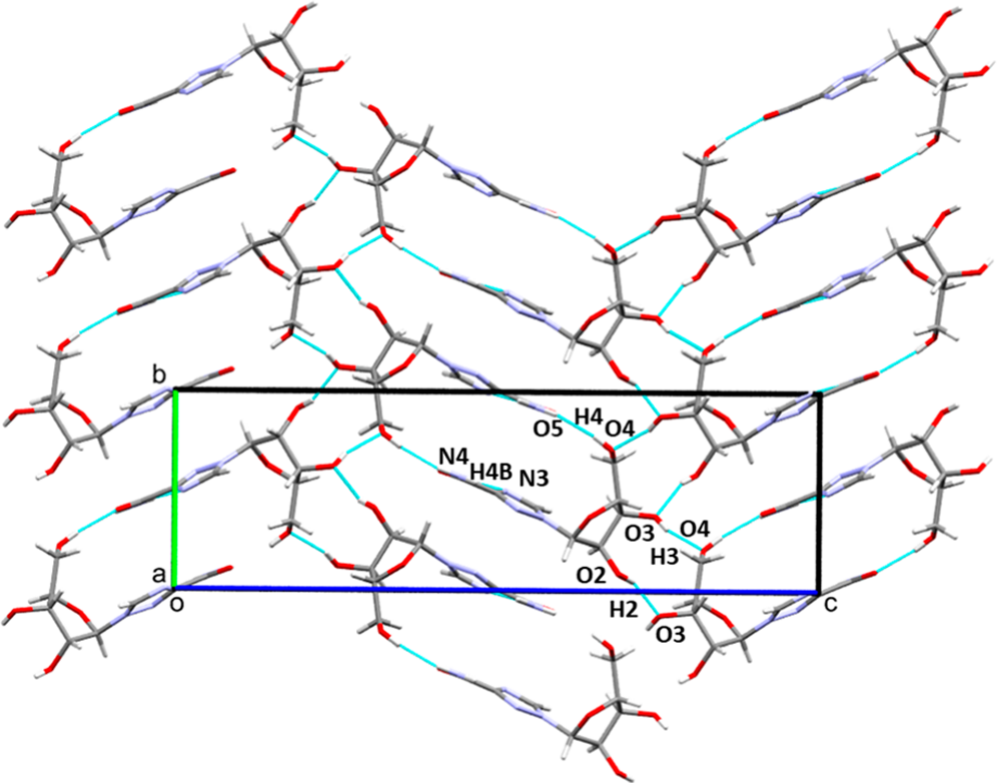
Packing arrangement of **V2** at ambient pressure viewed along the chains formed by interactions *A* and *C* (see text). The stacking produces a double layered herringbone structure characterized by strong H-bonding between the carboxamide and the sugar constituents in neighboring molecules. The packing features strong dispersion interactions between the stacked triazole rings.

### 3.2 The Response of V2 to Pressure

**V2** can be compressed to 5.5 GPa without undergoing any phase transitions. Fitting the pressure–volume (*P*–*V*) data to a third-order Birch–Murnaghan equation of state (Figure 4a) yields a bulk modulus *K*_0_ of 13.7(7) GPa and a pressure derivative *K′* of 9.2(1), with *V*_0_ fixed at the experimentally determined value of 1017.05(17) Å^3^. These values are similar to those reported in other H-bonded crystal structures, such as l-alanine (*K*_0_ = 13.1(6) GPa, *K*′ = 7.0(3)).^40^

**Figure 4:**
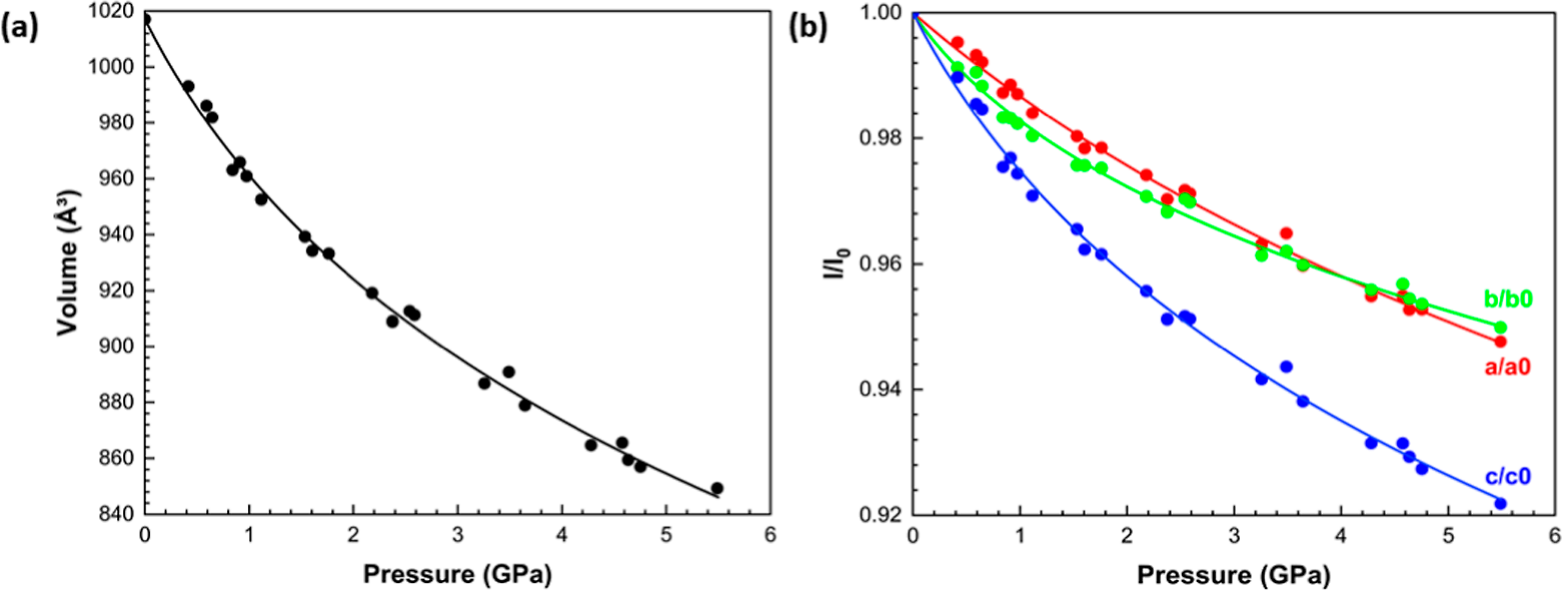
Variation of (a) the unit cell volume and (b) the normalized lattice cell parameters (*l*/*l*_0_) of the **V2** form as a function of pressure. 3rd order Birch–Murnaghan equations of state were fit to all data. Error bars are within the points.

The crystal structure of **V2** is orthorhombic and the principal values of the strain tensor are therefore aligned with the unit cell axes. Compression in the *ab* plane is approximately isotropic, with the *a* and *b*-axes decreasing in length by 5.2 and 5.0%, respectively (Figure 4b). The compression of the *c*-axis, which is somewhat higher (7.8%), changes the angle of the herringbone arrangement of double layered chains described above. The herringbone plane angle, measured as the angle between planes formed by the triazole rings in two molecules related by the operation [−*x* + 1/2, −*y* + 1, *z* – 1/2] in the supramolecular structure, increases from 55.2(5)° to 81.3(2)° from ambient pressure to 5.5 GPa (Figure S1).

The void space, which reduces from 234.3(4) Å^3^ to 101.1(1) Å^3^ between ambient pressure and 5.5 GPa, is predominantly aligned along the *c*-axis, which is also the most compressible direction. The furanosyl ring conformation changes at 0.4 GPa from E_2_ to the adjacent T_2_^1^ point in the pseudorotation pathway (Figures S2 and S3). The compression along the *b*-axis reflects the flexibility of the ribose unit which allows the hydroxymethyl and nucleobase moieties to twist by ∼6° about the C2–C1–N1–N2 torsion. This process begins to plateau at 3.0 GPa (Figure S4). This conformational form then persists across the remainder of the **V2** pressure series. The conformational change leads to a folding of the molecule, bringing the ribose and nucleobase groups into closer proximity, reducing its overall molecular volume from 203.5(2) Å^3^ to 202.0(2) Å^3^ (Figure S5).

PIXEL calculations were carried out at each pressure point and the total energies for each interaction are plotted in Figure 5. As pressure increases, interaction *A*/*A*′ initially stabilizes, but then begins to destabilize after 1.5 GPa. With the exception of the barely stabilizing interaction *F*, other interactions are destabilized with pressure. The effect is largest for the H-bonded interactions *D*, *E* and especially *C*, as OH···O H-bond angles deviate from linearity (e.g., from 168° to 151° for the O4H4···O5 angle in interaction *C*).^39^ The increase in repulsion can be explained by the approach of the hydroxymethyl and carboxamide groups in neighboring molecules, as the shortest distance between them (H4···O5) decreases by 0.06 Å, from ambient pressure to 5.5 GPa (Figure S6).

**Figure 5:**
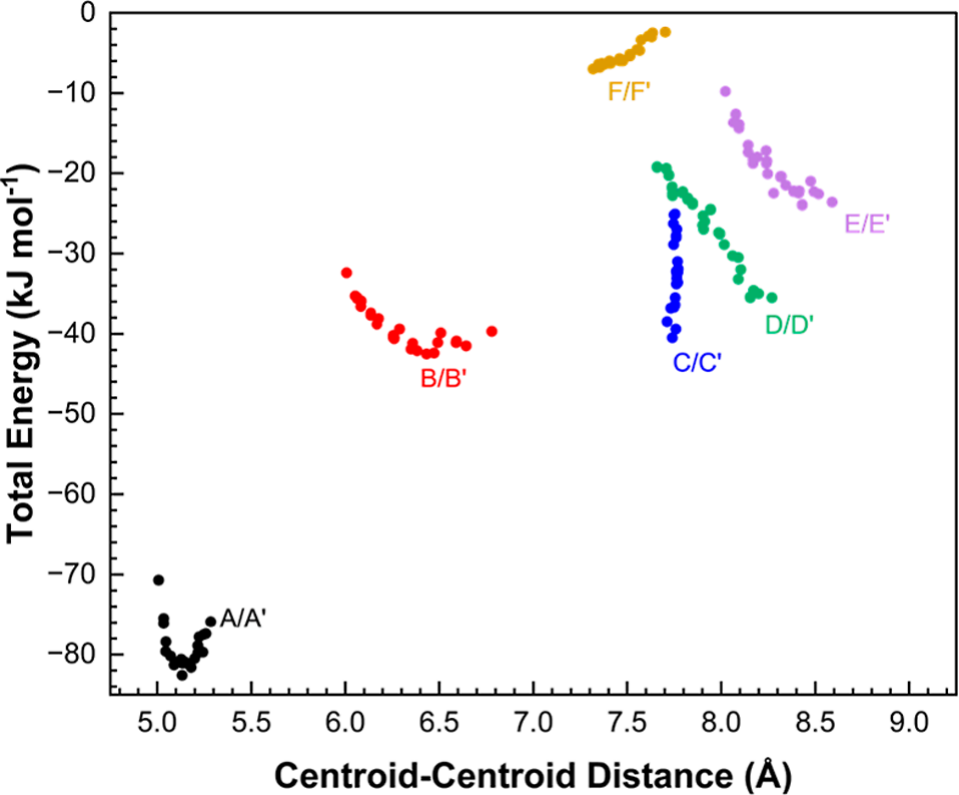
Intermolecular interaction energies of the first coordination sphere in the **V2** form as a function of centroid–centroid distance.

### 3.3 High-Pressure Phase Transitions

**V2** undergoes a phase transition to **V3** (*P*2_1_2_1_2_1_, *Z* = 12, *Z*′ = 3) between 5.3 and 6.0 GPa in which the length of the *b*-axis triples as the number of molecules in the asymmetric unit increases from 1 to 3. The transition pressure is approximately 5.3 GPa, though this is somewhat sample dependent. A breakdown of the six experimental runs is given in Table S2, and the phase sequences corresponding to each run number are shown in Figure S7. To rule out the possibility that the **V2**-to-**V3** transition was associated with the freezing of the neon PTM at 4.8 GPa, the study was repeated using nitrogen (freezing pressure of 2.4 GPa);^41^ **V3** was observed in both cases at similar pressures.

At 6.0 GPa and above, two phase pathways exist (Figure 6). In some samples, a transformation from **V3** to **V4** (*P*2_1_2_1_2_1_, *Z* = 4, *Z*′ = 1) occurs between 5.9 and 6.0 GPa, followed by a second transformation from **V4** to **V5** (*P*2_1_2_1_2_1_, *Z* = 4, *Z*′ = 1) between 7.0 and 7.2 GPa. In the second pathway, **V4** persists up to 10 GPa. Attempts to decompress samples were generally unsuccessful as crystal quality tended to deteriorate through the progression of phase transitions. In one case, decompression of **V5** at 7.2 GPa to ambient pressure was shown to regenerate **V2**, suggesting that the phase transitions listed are reversible.

**Figure 6:**
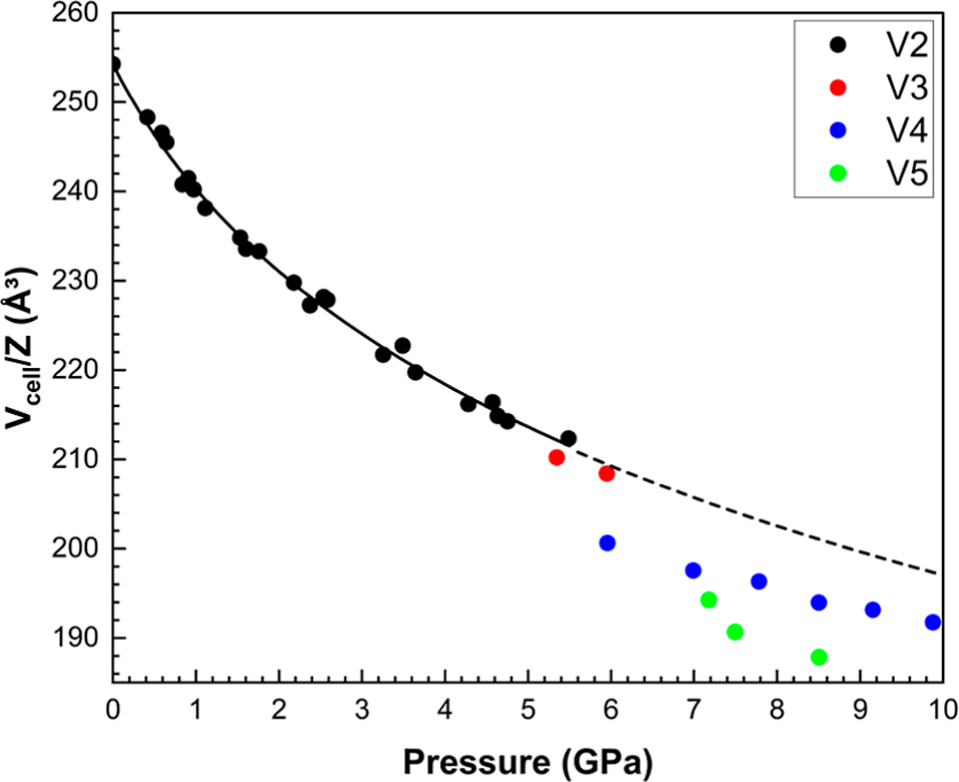
Plot of molecular volume divided by the asymmetric unit as a function of pressure. The dashed line is the extrapolated 3rd order Birch–Murnaghan equation fit to the **V2** volumes.

The sequence of transitions may be influenced by kinetic effects, with both transition pressures and phase sequences varying between samples. Indeed, in one loading, indexing of data collected at 6.1 GPa showed that **V3** and **V4** can coexist in the same crystal (Figure S8), though poor data quality prevented integration of the two domains. The unit cell volume divided by the number of molecules in the unit cell, (*V*_cell_/*Z*) decreases at each transition (Figure 6). Extrapolation of the equation of state of **V2** to 10 GPa demonstrates the significantly more efficient packing as result of the sequence of transitions. There is a relatively small decrease in *V*_cell_/*Z* at the **V2**-to-**V3** transition (0.8 Å^3^ for **V2** at 5.49 GPa and **V3** at 5.35 GPa). In contrast, the decrease between **V3** and **V4** is substantially greater, with a difference of 2.0 Å^3^ between the volume of **V3** at 5.95 GPa and that of **V4** at 5.96 GPa. Overall, the *PV* contribution to the Gibbs free energy difference between **V2** and **V5** at 8.5 GPa is −68.9 kJ mol^–1^, an enormous figure in the context of typical overall free energy differences between polymorphs of organic materials.^42^

The cubic close packed (ccp) topology described above for phase **V2** persists at all pressures and in all phases. The bond lengths and angles retain typical values, and the changes in the intermolecular contacts that define the new high-pressure forms are related to conformational changes of the furanosyl ring. All three transitions are accompanied by changes in the conformations of the ribose units while that of the nucleobase remains the same throughout. The conformational changes occur via rotations of the hydroxymethyl group and by pseudorotation of the furanosyl rings, in which different atoms move in and out of the mean plane of the ring. Conformational descriptors for each phase are summarized in Table 3. The **V2** and **V3** forms are closely related both in their packing and the relative orientations of the ribose and the nucleobase. The furanosyl ring conformation moves anticlockwise along the Altona pseudorotation pathway (Figure 7a,b) in **V2** and even after the phase transition to **V3**, molecule 2 retains the T_2_^1^ conformation. Molecule 1 adopts the neighboring E^1^ conformation, while molecule 3 adopts a T_2_^3^ conformation, which is three steps away on the pseudorotation pathway. The distinct conformational difference leads to molecules 1 and 2 forming hydrogen bonded pairs while molecule 3 pairs with a symmetry equivalent of itself.

**Table 3:** Selected Data of Pseudorotation Angles and Ring Conformation Descriptors for all Forms of Ribavirin Discussed in This Work^a^

pressure (GPa)	phase	*P* (°)	descriptor	shorthand notation
0	**V2**	336.0(4)	E C2-*exo*	E_2_
5.49	**V2**	320.1(4)	T C1-*endo*, C2-*exo*	T_2_^1^
5.35 (Mol. 1)	**V3**	312.4(7)	E C11-*endo*	E^1^
5.35 (Mol. 2)	**V3**	320.3(6)	T C21-*endo*, C22-*exo*	T_2_^1^
5.35 (Mol. 3)	**V3**	6.9(7)	T C32 -*exo*, C34 -*endo*	T_2_^3^
5.96	**V4**	150.4(6)	T C1-*exo*, C2-*endo*	T_1_^2^
7.18	**V5**	81.7(7)	E O1-*endo*	E^0^

a A complete table for each dataset is given in Table S3.

**Figure 7:**
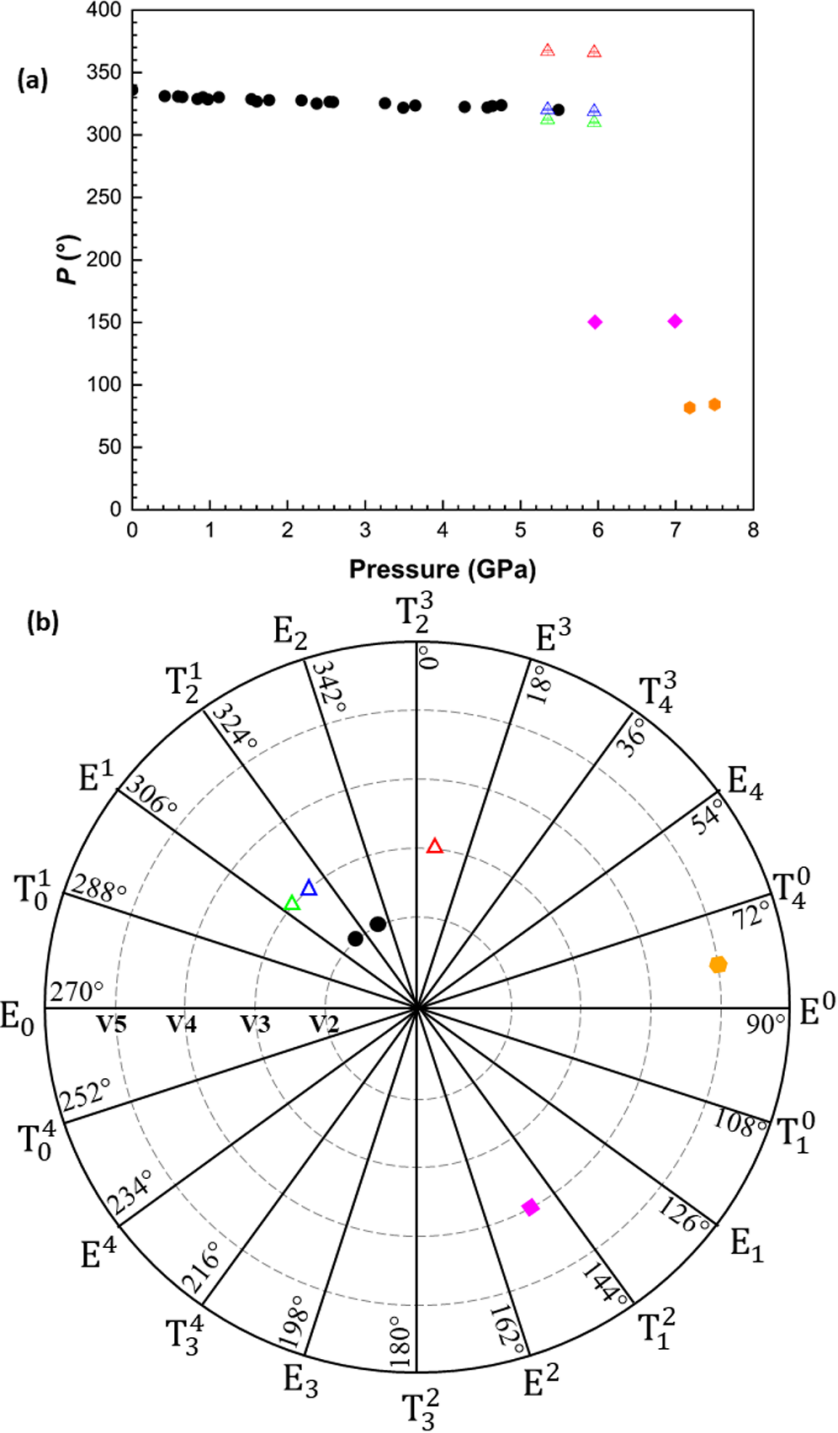
(a) Variation in pseudorotation (*P*) angle of all structures of all phases (**V2:** black circles, **V3:** triangles colored by symmetry equivalence, **V4:** diamonds, **V5:** hexagons) with respect to pressure. Complementary angles are plotted above 360° for ease of reading. (b) *P* angles of data from Table 3 plotted on a pseudorotation pathway.

Phases **V4** and **V5** exhibit considerable differences in pseudorotation angles and occupy very different positions along the pseudorotation pathway with respect to **V2** (Figure 7 ). Differences in molecular conformations and crystal packing are shown in Figure 8. The furanosyl ring in **V4** adopts a T_1_^2^ conformation (as opposed to the T_2_^1^ conformation seen in **V2** and in molecule 2 of **V3**) while in **V5** it adopts an E^0^ conformation. The **V2** and **V4** forms differ by ∼170° in pseudorotation angle, resulting in marked changes in the torsions about the glycosidic bond (Figure S9a). Between 5.5 and 6.0 GPa, the C2–C1–N1–N2 and C2–C1–N1–C6 torsion angles change from −171.7(4)° and −57.1(5)° in **V2** to 167.7(7)° and −2.9(12)° in **V4**. The conformational changes also alter the orientations of the hydroxyl groups based on O2 and O3, while the hydroxymethyl moiety exhibits its own torsional flexibility as the O4 atom is not adjacent to the ring. This is further demonstrated in the transition from **V4** (T_1_^2^) to **V5** (E^0^), which occurs over a narrow pressure range (7.0 to 7.2 GPa) in which the C4–C5–C7–O4 torsion angle changes from 52.8(11)° in **V4** to 38.9(9)° in **V5** (Figure S9b). Otherwise, the conformation of the molecule in **V5** is very similar to that in **V4**, with minimal deviation in the positions of the nucleobase atoms (RMSD = 0.05 Å). The **V4**-to-**V5** transition is accompanied by a small decrease in molecular volume, with *V*_cell_/*Z* for **V4**-to-**V5** decreasing by 3.3 Å^3^ between the 7.0 and 7.2 GPa structures.

**Figure 8:**
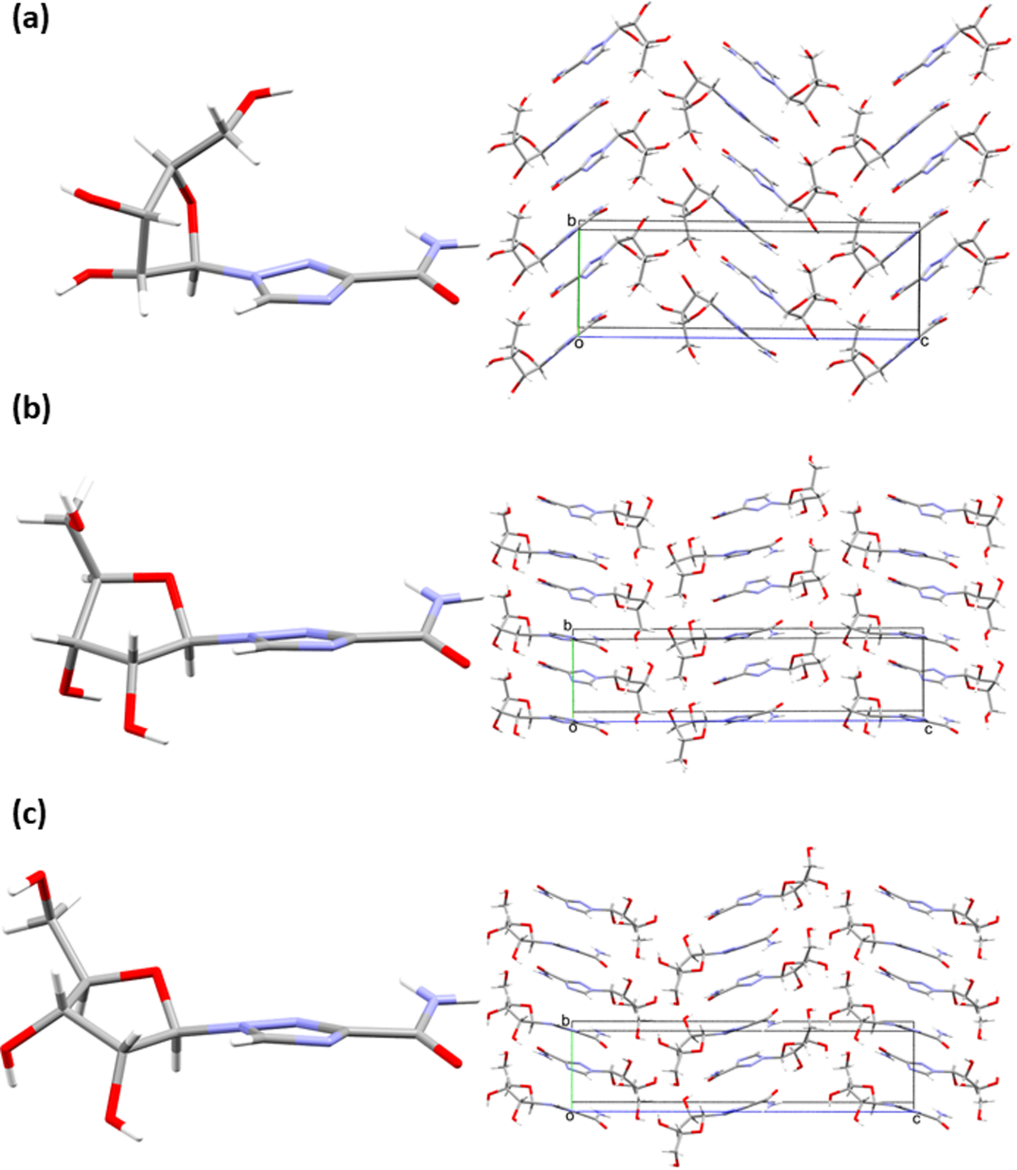
Asymmetric unit and crystal packing diagrams of (a) the **V2** form at ambient pressure, (b) the **V4** form at 6.0 GPa and, (c) the **V5** form at 7.0 GPa.

The conformational changes that occur over the course of the phase transitions alter the orientations of the hydroxyl substituents, leading to reorganization of the hydrogen bonding and thereby the energetic hierarchy of the intermolecular interactions. In **V4**, there are three hydrogen bonds which increases to five in **V5** as interaction *E* involves two additional hydrogen bonds. PIXEL calculations and the hydrogen bonds for each high-pressure structure are given in the Supporting Information.

### 3.4 V3

The **V2** to **V3** phase transition was observed in two separate runs, one at 5.3 GPa and another at 6.0 GPa. In a third run, a transition from **V2** to a mixed **V3/V4** phase was observed at 6.1 GPa (see Table S2 and Figure S7). The naming scheme in this new phase has 1, 2, or 3 prefixed to the atomic numbering of **V2** to indicate its residue number (Figure 9a). **V3** shares the same double herringbone packing motif of **V2** but the sequence of molecules 1, 2, and 3 are distributed along the *b*-axis in the sequence 1–1–2–3–3–2 (Figure 9b).

**Figure 9:**
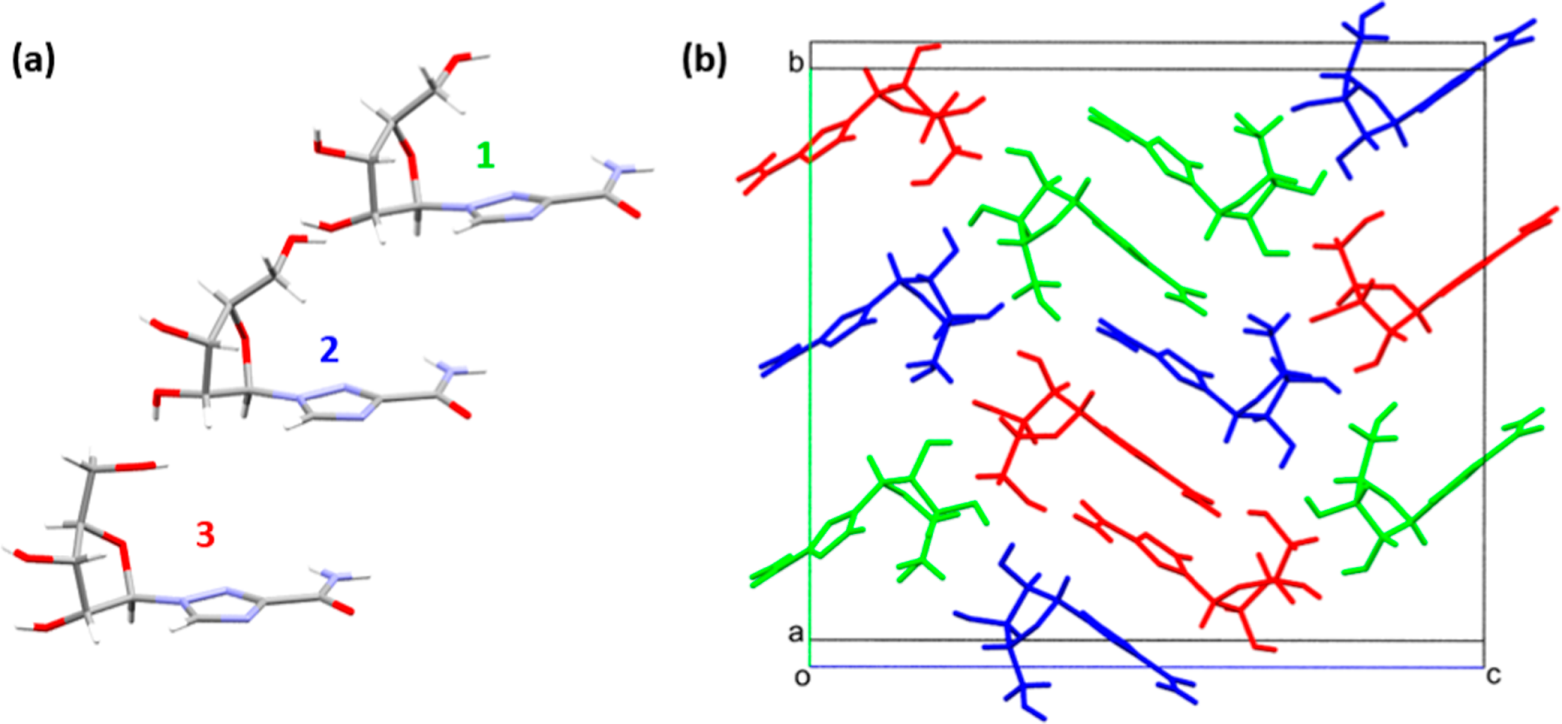
(a) The asymmetric unit and (b) the unit cell of the **V3** form. The inequivalent molecules in the unit cell are colored 1 green, 2 blue, and 3 red.

The RMSDs of the non-H positions in molecules 1, 2, and 3 in **V3** compared to those in **V2** are 0.11, 0.06, and 0.52 Å, respectively, indicating that the **V2**-to-**V3** transition occurs as a result in the change in the conformation of molecule 3. The conformation of the ribose unit in molecule 3 closely resembles that in the other known ambient conditions polymorph **V1** (reference code VIRAZL, RMSD = 0.07 Å), although the orientations of the base moiety are different (Figure S10).

### 3.5 V4

The sense of the twisted T_2_^1^ furanosyl conformation in **V2** flips in **V4** to become T_1_^2^, resulting in molecules which are significantly flatter in projection along the *a* direction. This compresses the stacking distance of the rectangular motif referred to in Section 3.1, causing the *b*-axis length to decrease by 15% and the *c*-axis length to increase by 13%. Interactions *C*/*C*′ stabilize by comparison with **V2** as a result of the change in the stacking distance, while interaction *A*/*A*′ destabilize significantly.

While the overall arrangement of the molecules in the first coordination sphere is unchanged, there is a reorganization in the energy ranking of the contacts (Figure 10) as the flip in the furanosyl conformation replaces the hydrogen bond network present along the *c*-axis in **V2** (Figure 4) with one between molecules stacked along the *a*- and *b*-axes involving O2H2···O3 and O3H3···O4. The loss of H-bonds occurs in interactions *D* and *E* which are therefore substantially weakened (Tables S4 and S5, Figure 10).

**Figure 10:**
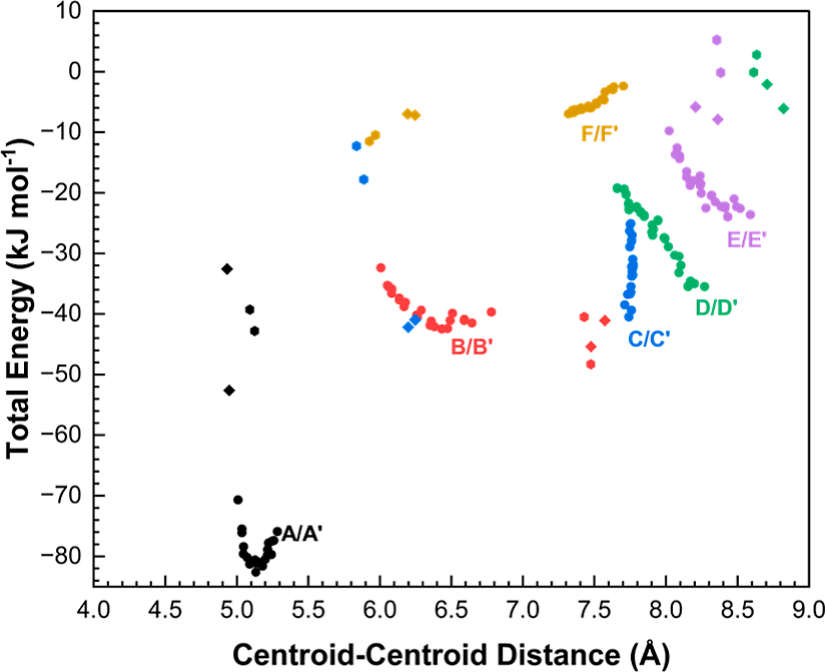
Total energy of each interaction present in the first coordinate sphere plotted against centroid–centroid distance, for all phases. **V2**, **V4**, and **V5** contacts are indicated by circles, diamonds, and open hexagons, respectively. Tables S4–S7 break down the energies for each interaction present in **V4** and **V5**.

The modified H-bond network changes the herringbone angle described in Section 3.2 from 81.3(2)° (**V2** at 5.5 GPa) to 19.9(4)° (**V4** at 6.0 GPa), or 24.2(4)° (**V4** at 7.0 GPa), with the “flattening” of the motif reflecting the flattening of the molecular conformation (Figure S11).

The position of the hydroxyl H3 atom differs markedly between the **V4** structures determined at 6.0 and 7.0 GPa in separate experimental runs, with the C2–C4–O3–H3 torsion angle varying by 151°. To confirm this unusually large difference, models with alternative torsional angles were optimized using periodic DFT: the H3 position observed at 7.0 GPa was tested in the 6.0 GPa structure, and vice versa. In both cases, the experimentally assigned positions were found to be ∼ 4 kJ mol^–1^ lower in energy, validating the apparent change in orientation.

### 3.6 V5

It is clear from Figure 10 that while **V2** and **V4** are very different, the changes occurring across the **V4** to **V5** transition are more modest Figure S9b). By far the biggest energetic change is destabilization of interaction *C* as the result of the lengthening of N4H4A···O4 from 2.02 Å in **V4** to 2.53 Å in **V5**, reducing the electrostatic energy by 23 kJ mol^–1^. Interaction *E* forms two hydrogen bonds, O4H4···O3 and O3H3···O3, but the strong Coulombic energies are diminished by a large repulsion component thereby reducing the overall strength of the interaction (Tables S4 and S5). As was the case in **V4**, this change in H-bonding is the result of a conformational change in the furanosyl ring, from T_1_^2^ in **V4** to E^0^ in **V5** which modifies the orientation of the hydroxymethyl group of which O4 is part. This allows still further reduction in the triazole stacking distances in the rectangular motif from 4.41(1) Å to 4.25(1) Å.

## 4 Conclusion

We have studied the effects of pressure on the **V2** form of ribavirin up to 10 GPa, with crystal structures refined to 7.5 GPa and cell dimensions determined thereafter. We show that the adoption of alternative conformations by the flexible furanosyl ring in ribavirin results in changes in the underlying hydrogen-bond network, driving high-pressure phase transitions. The three new high-pressure phases (**V3**, **V4**, and **V5**) identified within a narrow pressure range (5–7 GPa) highlight the phase diversity that can arise from the inclusion of flexible groups in small molecules. This idea might extend to other furanose containing molecules, such as other nucleoside analog APIs, which may have implications in drug design or polymorph discovery.

The transition from **V2** to **V3** leads to the adoption of a new conformation in one of the three unique molecules in the asymmetric unit. The ribose and nucleobase residues in the new conformation resemble those in the ambient-pressure **V1** polymorph, but their relative orientation about the glycosidic bond differs. **V3** transforms into **V4** above 6 GPa. Although the topology of the packing remains unchanged, **V4** adopts a completely new molecular conformation, altering the hierarchy of the intermolecular interaction energies. Another, more minor, conformational change leads to **V5** above 7 GPa. The low energy barrier between twist and envelope ring conformations facilitates these transitions.

## Supplementary Material



## Footnotes

1 Sub- and superscript notation follows Altona and Sundaralingam's work, and signifies which atom lies above (i.e., on the same side of the ring as the CH_2_OH group) or below the mean plane of the ring, respectively. It does not follow the crystallographic atomic numbering of the molecule but rather goes around the ring consecutively starting with oxygen which is denoted as 0 following the sequence O0-C1-C2-C3-C4 which equates to O1−C1−C2−C4−C5 used for crystal structure labeling. The parameter *P* represents the phase of the pseudorotation of the five-membered ring and is calculated from the five torsion angles formed about the bonds. See ref. 9 for details..

## Notes

Crystallographic data (CIFs), tables, figures (PDF)
