# Supplementary material for: High-Pressure Polymorphism of Ribavirin
Source: Cryst Growth Des. 2025 May 12;25(10):3537–47. doi: 10.1021/acs.cgd.5c00372 (PMC12100640; doi:10.1021/acs.cgd.5c00372)
Supplement: Supplementary file 1 [file cg5c00372_si_001.pdf]

SUPPLEMENTARY MATERIAL

High-pressure polymorphism of ribavirin

Bhaskar Tiwari[1,2], Hanns-Peter Liermann[2], Simon Parsons*[1], Nico Giordano*[2]

1. *Centre for Science at Extreme Conditions, School of Chemistry, The University of Edinburgh EH9 3FJ, Edinburgh, U.K.*
2. *Deutsches Elektronen-Synchrotron DESY, Notkestr. 85, 22607 Hamburg, Germany*

*Corresponding Authors: simon.parsons@ed.ac.uk; nico.giordano@desy.de

Electronic Supplementary Information Table of Contents

Table S1………………………………………………………………………………..3

Figure S1………………………………………………………………………………8

Figure S2………………………………………………………………………………8

Figure S3………………………………………………………………………………8

Figure S4………………………………………………………………………………9

Figure S5………………………………………………………………………………9

Figure S6……………………………………………………………………………..10

Figure S7……………………………………………………………………………..10

Figure S8……………………………………………………………………………..11

Figure S9……………………………………………………………………………..11

Figure S10……………………………………………………………………………12

Figure S11……………………………………………………………………………12

Figure S12……………………………………………………………………………13

Figure S13……………………………………………………………………………13

Table S2………………………………………………………………………………14

Table S3………………………………………………………………………………15

Table S4………………………………………………………………………………16

Table S5………………………………………………………………………………17

Table S6………………………………………………………………………………18

Table S7………………………………………………………………………………19

Table S1: Summary of all crystal structure and refinement data for all **V2-V5** structures

**Pressure (GPa)**	**0.00**	**0.42**	**0.59**	**0.65**	**0.84**	**0.91**
**Phase**	V2	V2	V2	V2	V2	V2
**Crystal Data**						
a, b, c **(Å)**	5.2848(5), 7.7035(8), 24.982(2)	5.2599(3), 7.6361(13), 24.726(4)	5.2493(2), 7.6306(12), 24.6192(18)	5.24328(13), 7.6136(7), 24.5977(14)	5.2175(2), 7.5751(11), 24.368(2)	5.2243(1), 7.5753(10), 24.4047(12)
V **(Å³)**	1017.05(17)	993.1(2)	986.13(18)	981.94(11)	963.10(17)	965.83(14)
**Z**	4	4	4	4	4	4
**Data Collection**						
**No. of measured, independent and observed [*I* > 2σ(*I*)] reflections**	14610, 2083, 1318	2432, 1203, 1158	2396, 1162, 859	2196, 1143, 1018	2442, 1171, 1104	2414, 1082, 1032
R_{int}	0.087	0.023	0.046	0.037	0.023	0.020
**Refinement**						
$R[F^2 > 2\sigma(F^2)]$, $wR(F^2)$, S	0.053, 0.153, 1.02	0.031, 0.090, 0.98	0.045, 0.094, 1.14	0.046, 0.137, 1.19	0.030, 0.084, 1.17	0.048, 0.233, 1.13

Pressure (GPa)	0.98	1.10	1.50	1.60	1.80	2.18
**Phase**	V2	V2	V2	V2	V2	V2
**Crystal Data**						
***a, b, c* (Å)**	5.21634(14), 7.5676(6), 24.3419(19)	5.2003(3), 7.5524(12), 24.254(2)	5.1808(2), 7.5163(12), 24.121(2)	5.17060(13), 7.5159(6), 24.0408(13)	5.17110(14), 7.5128(9), 24.0212(11)	5.1481(1), 7.4782(10), 23.8755(12)
**V (Å^3)**	960.90(11)	952.57(18)	939.30(18)	934.27(9)	933.21(12)	919.17(13)
**Z**	4	4	4	4	4	4
**Data Collection**						
**No. of measured, independent and observed [$I > 2\sigma(I)$] reflections**	2348, 1163, 1128	2506, 1111, 1038	2461, 1088, 1025	2126, 1101, 1034	2438, 1077, 1033	2258, 1014, 959
**R_{int}**	0.023	0.030	0.026	0.025	0.028	0.020
**Refinement**						
**$R[F^2 > 2\sigma(F^2)]$, $wR(F^2)$, S**	0.031, 0.094, 1.18	0.035, 0.092, 1.17	0.030, 0.079, 1.14	0.037, 0.099, 1.18	0.035, 0.089, 1.16	0.047, 0.214, 1.23

**Pressure (GPa)**	**2.38**	**2.38**	**2.54**	**2.60**	**3.30**	**3.49**
**Phase**	V2	V2	V2	V2	V2	V2
**Crystal Data**						
a, b, c **(Å)**	5.12795(16), 7.4600(9), 23.7653(15)	5.12792(14), 7.4589(6), 23.7624(18)	5.1355(2), 7.4753(10), 23.7753(15)	5.13275(15), 7.4712(12), 23.7648(17)	5.09005(16), 7.406(1), 23.5241(13)	5.0992(1), 7.4111(9), 23.5732(11)
V **(Å³)**	909.13(13)	908.88(10)	912.72(14)	911.32(16)	886.78(14)	890.85(12)
**Z**	4	4	4	4	4	4
**Data Collection**						
**No. of measured, independent and observed [*I* > 2σ(*I*)] reflections**	1875, 1038, 980	2202, 1095, 1061	2244, 1126, 950	2196, 1027, 987	2330, 1008, 941	2152, 988, 927
R_{int}	0.029	0.023	0.035	0.021	0.019	0.019
**Refinement**						
$R[F^2 > 2\sigma(F^2)]$, $wR(F^2)$, S	0.031, 0.088, 1.17	0.028, 0.076, 1.14	0.039, 0.087, 1.14	0.032, 0.084, 1.21	0.029, 0.082, 1.16	0.044, 0.198, 1.21

Pressure (GPa)	3.65	4.28	4.58	4.64	4.80	5.49
**Phase**	V2	V2	V2	V2	V2	V2
**Crystal Data**						
a, b, c **(Å)**	5.0719(2), 7.3942(11), 23.436(2)	5.04644(9), 7.3637(4), 23.2703(10)	5.0467(2), 7.3709(11), 23.2693(18)	5.03506(19), 7.3528(9), 23.2157(18)	5.03531(18), 7.3466(11), 23.1680(17)	5.2848(5), 7.7035(8), 24.982(2)
V **(Å³)**	878.91(16)	864.73(6)	865.60(15)	859.48(13)	857.04(14)	843.99(16)
**Z**	4	4	4	4	4	4
**Data Collection**						
**No. of measured, independent and observed [$I >$ 2σ(I)] reflections**	2202, 1059, 997	2108, 1041, 1000	2006, 1073, 963	1961, 1008, 922	14610, 2083, 1318	1993, 1010, 966
R_{int}	0.029	0.023	0.033	0.028	0.087	0.023
**Refinement**						
$R[F^2 > 2\sigma(F^2)]$, $wR(F^2)$, S	0.037, 0.116, 1.11	0.029, 0.081, 1.18	0.032, 0.078, 1.17	0.034, 0.092, 1.20	0.053, 0.153, 1.02	0.030, 0.083, 1.12

**Pressure (GPa)**	**5.35**	**5.95**	**5.96**	**6.99**	**7.18**	**7.50**
**Phase**	V3	V3	V4	V4	V5	V5
**Crystal Data**						
a, b, c **(Å)**	5.0234(2), 21.287(3), 23.590(2)	5.01372(11), 21.238(2), 23.4880(8)	4.9465(3), 6.2449(10), 25.978(2)	4.9317(5), 6.1935(16), 25.870(5)	5.1260(3), 5.970(2), 25.389(3)	5.0907(3), 5.9279(11), 25.274(2)
V **(Å³)**	2522.6(5)	2501.0(3)	802.46(16)	790.2(3)	777.0(3)	762.71(16)
**Z**	12	12	4	4	4	4
**Data Collection**						
**No. of measured, independent and observed [$I > 2\sigma(I)$] reflections**	5307, 2847, 2330	12886, 6393, 4836	1999, 951, 863	1577, 898, 763	1451, 749, 650	1825, 883, 676
R_{int}	0.048	0.053	0.038	0.064	0.031	0.064
**Refinement**						
$R[F^2 > 2\sigma(F^2)]$, $wR(F^2)$, S	0.044, 0.100, 1.00	0.065, 0.270, 1.03	0.041, 0.111, 1.15	0.068, 0.194, 1.06	0.045, 0.124, 1.05	0.072, 0.204, 1.05

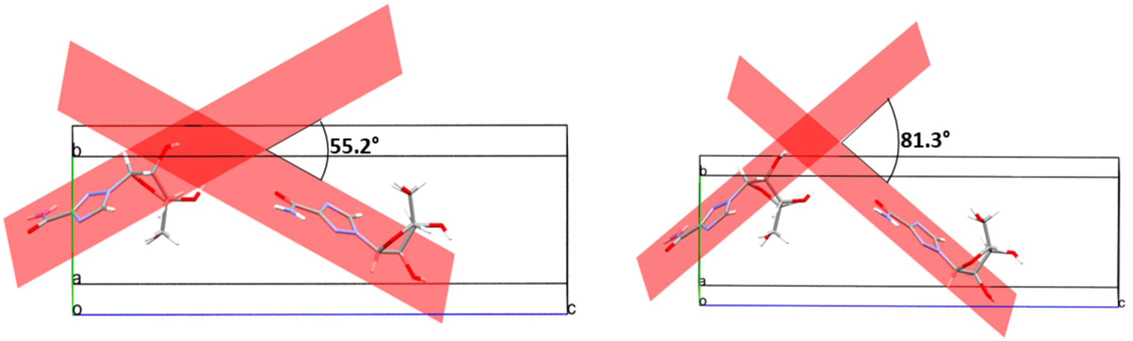

Figure S1 Herringbone angle made by two ribavirin molecules at ambient pressure and at 5.49 GPa. The planes are calculated based on the triazole moiety which is stable throughout the **V2** pressure series.

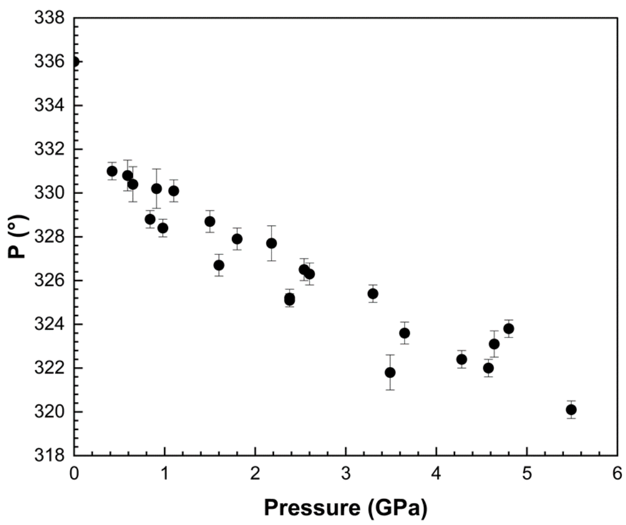

Figure S2 Pseudorotation angle, *P*, of the furanosyl ring as a function of pressure for **V2**.

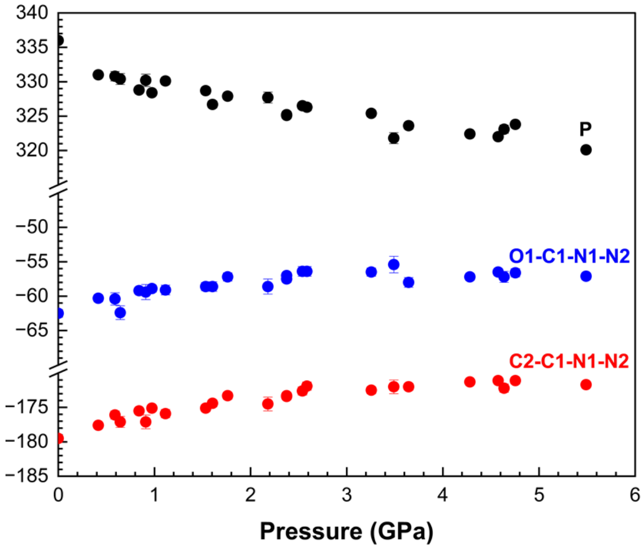

Figure S3 Relation between the pseudorotation angle, *P*, (black) and the C2−C1−N1−N2 (red) and O1−C1−N1−N2 (blue) torsion angles. The red and blue points show how the nucleobase folds into the available space.

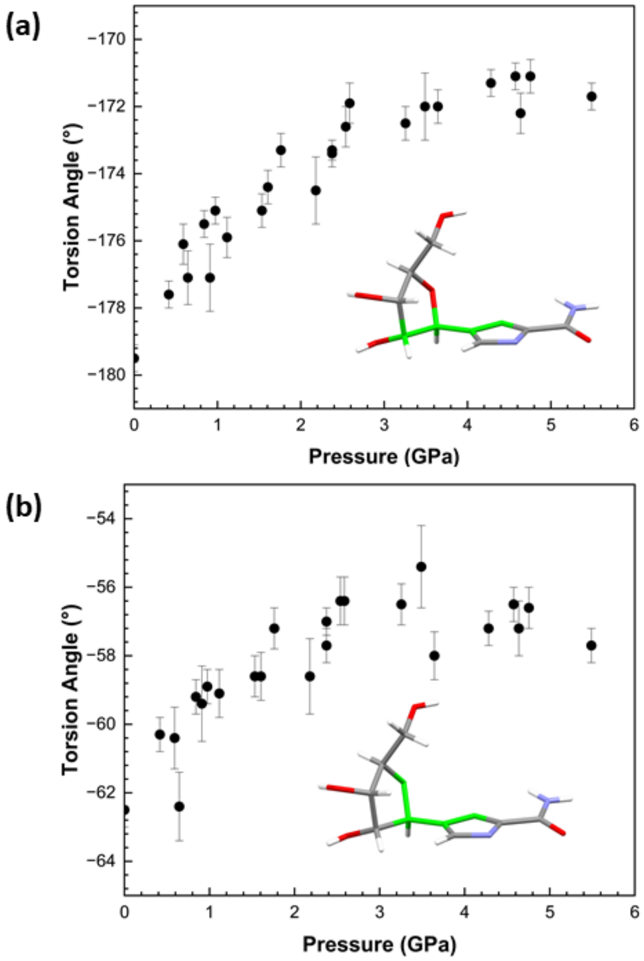

Figure S4 Twisting of the **(a)** C2−C1−N1−N2 and **(b)** O1−C1−N1−N2 torsion angles in **V2** with respect to pressure.

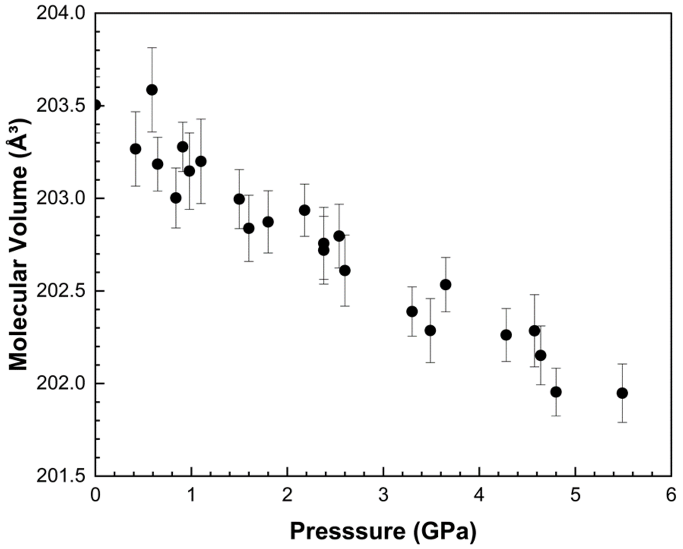

Figure S5 Change in molecular volume for the V2 series calculated using the MolVol program, which uses a Monte-Carlo algorithm to calculate the molecular volume. Each calculation used 8 million points and the largest error is 0.11%.

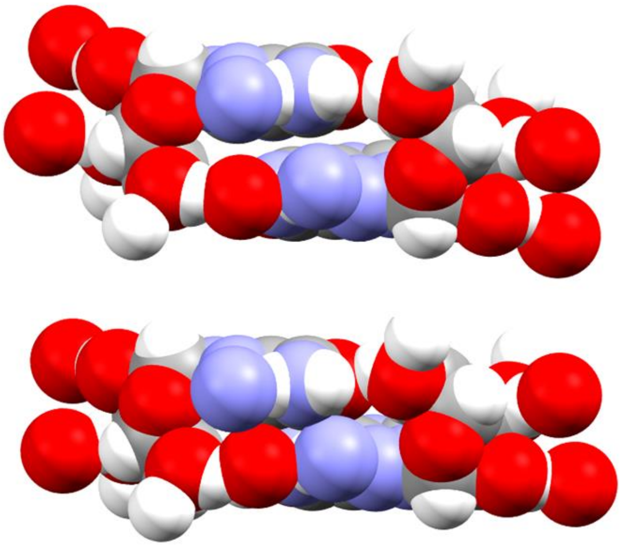

Figure S6 Change in void space between the central reference molecule and contact C at ambient pressure (top) and at 5.5 GPa (bottom).

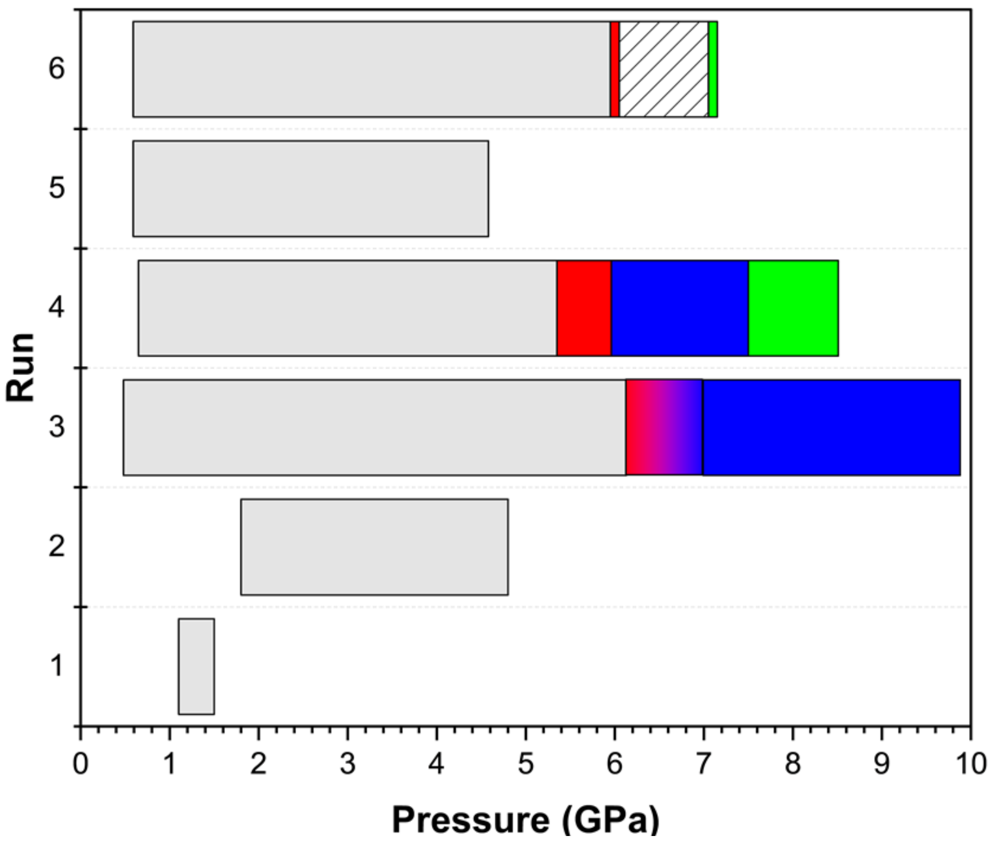

Figure S7 Bar chart illustrating the pressure ranges over which data were collected for each experimental run. Single-phase regions are indicated by the following color scheme: **V2** (grey), **V3** (red), **V4** (blue), and **V5** (green). In run 3, a red-to-blue gradient denotes a mixed phase region where data were obtained. In run 6, black diagonal lines represent the anticipated location of the **V4** phase, corresponding to a pressure interval that was missed during data collection.

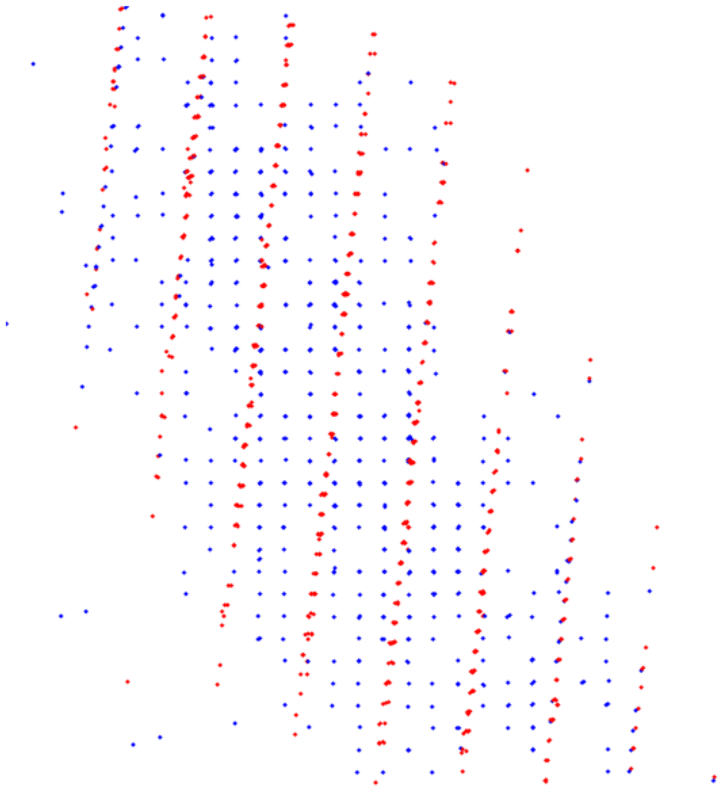

Figure S8 Reciprocal lattice space projection along the *b*-axis showing **V3** (blue) and **V4** (red) co-existing at 6.1 GPa.

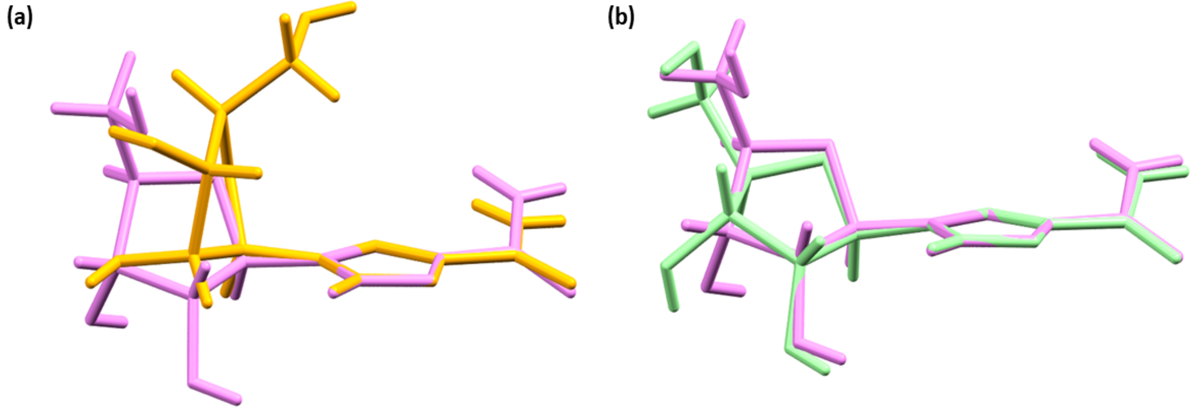

Figure S9 Structure comparisons of asymmetric units of **(a) V2** (orange) and **V4** (purple) and **(b) V4** (purple) and **V5** (green) structures.

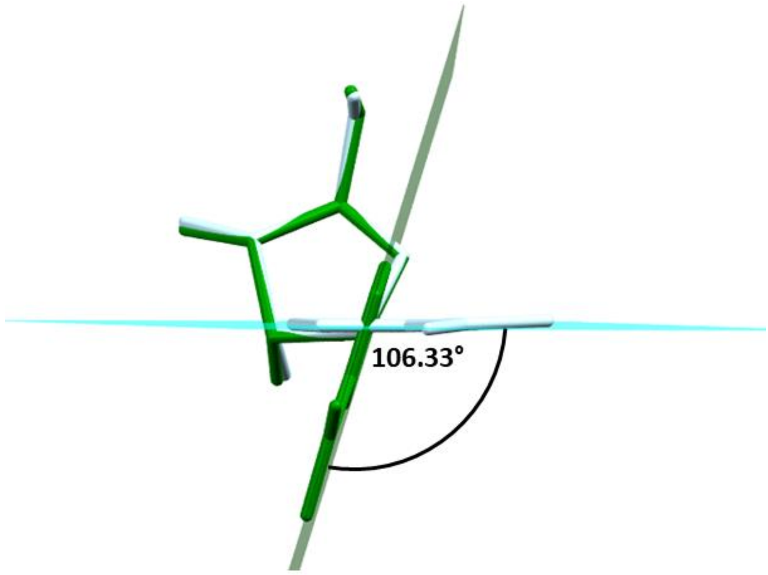

Figure S10 Rotation angle of the nucleobase along the glycosidic bond between **V1** at ambient pressure (green) and molecule 3 in **V3** (blue). The ribose substituents are very close matches as shown by the overlay.

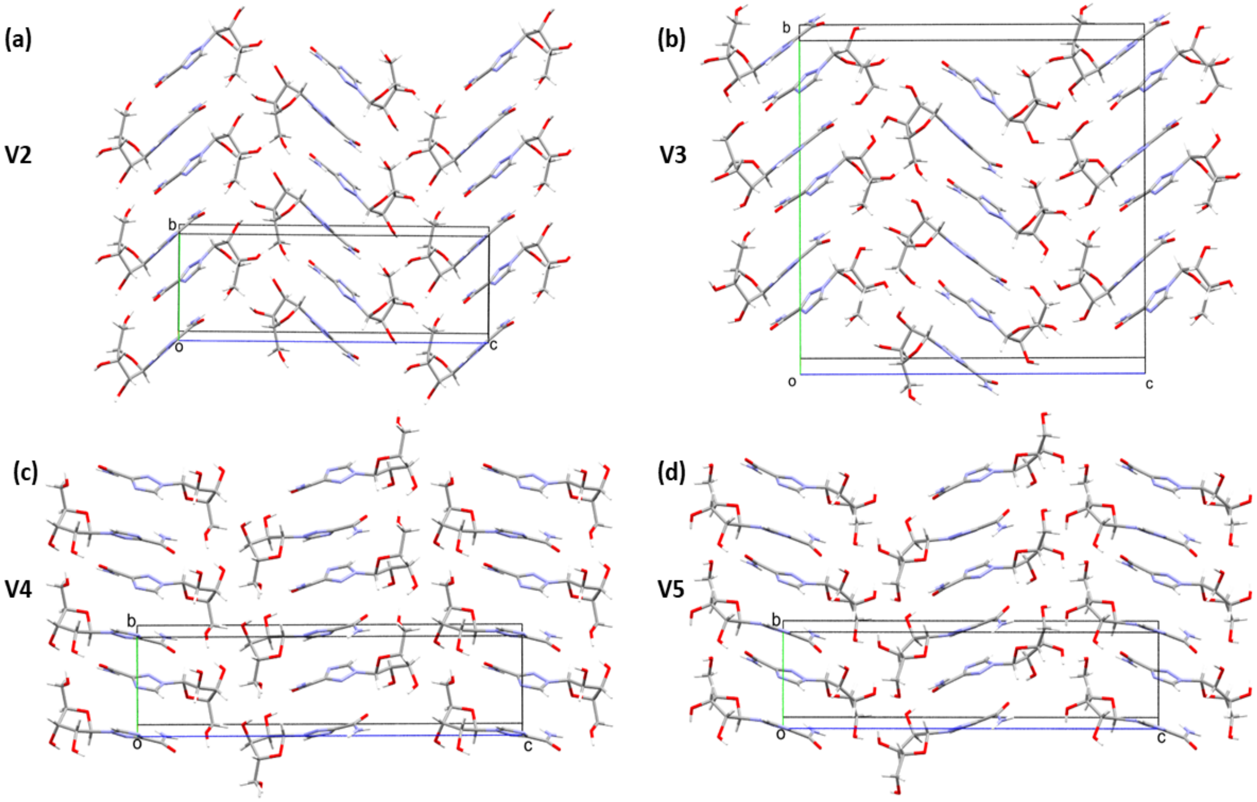

Figure S11 Packing diagrams viewed down the *a*-axis of **(a) V2** at 5.5 GPa **(b) V3** at 5.3 GPa **(c) V4** at 6.0 GPa and **(d) V5** at 7.0 GPa

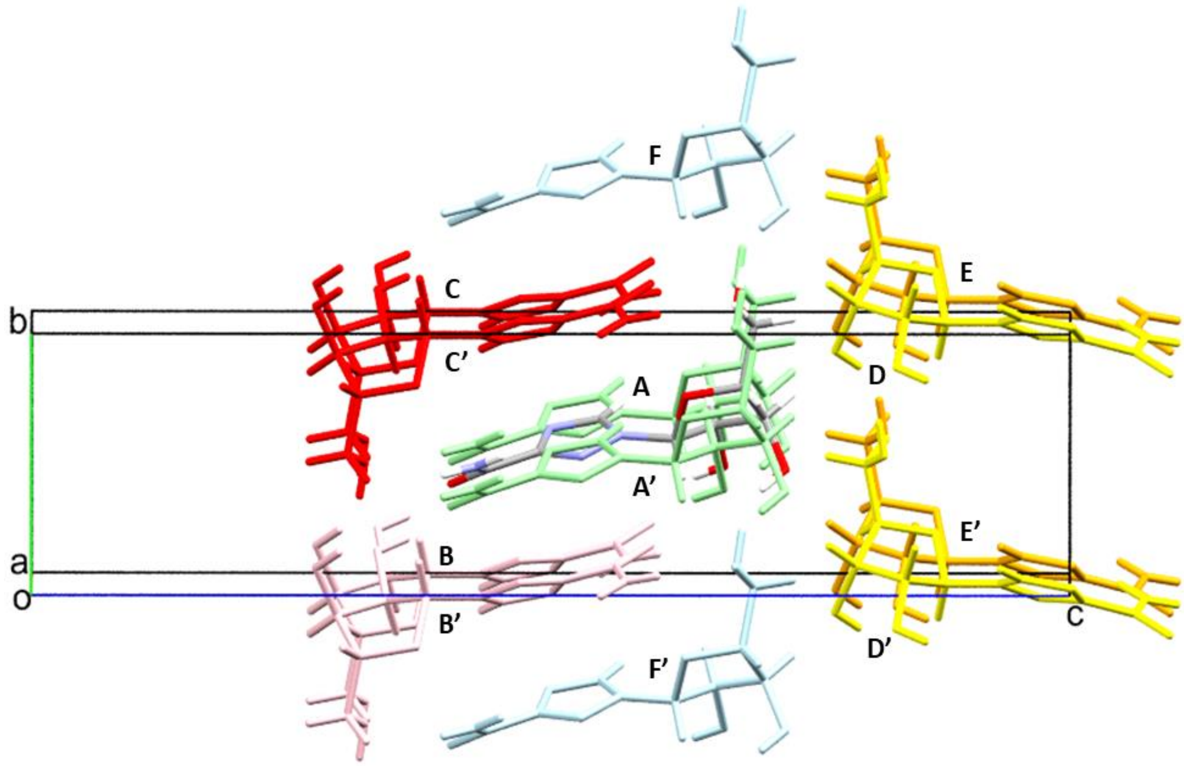

Figure S12 First coordination sphere of the **V4** form at 6.0 GPa.

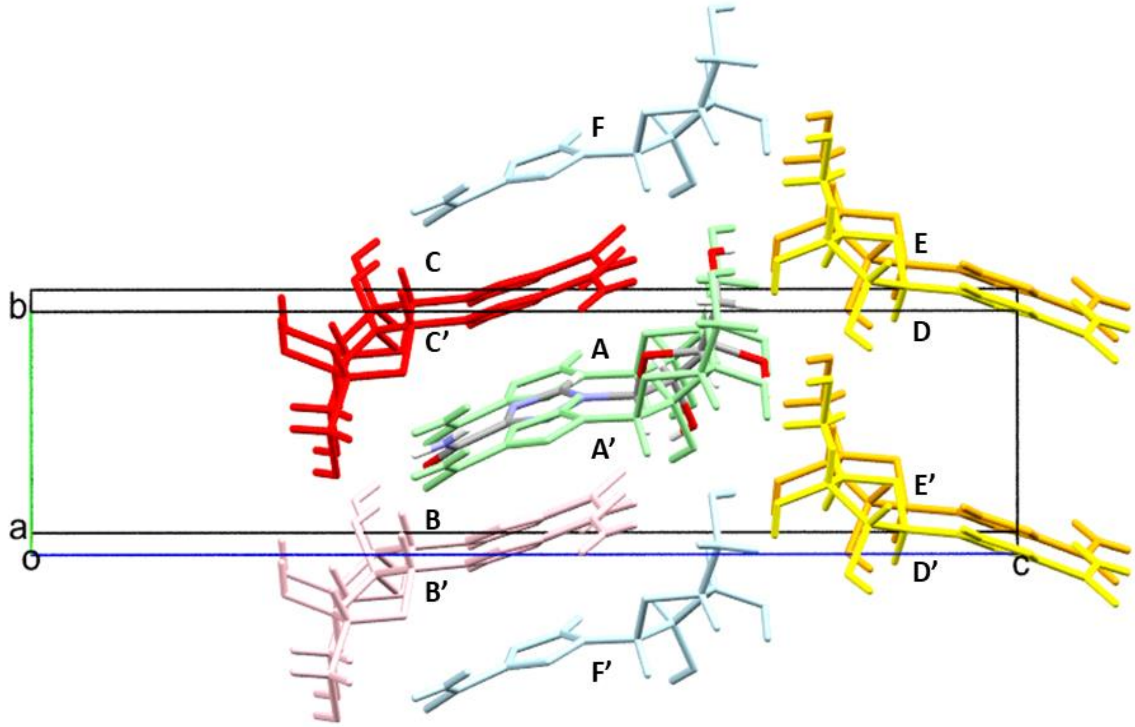

Figure S13 First coordination sphere of the **V5** form at 7.2 GPa.

Table S2: Breakdown of each experimental run in this study. Including pressure point and step, pressure transmitting medium (PTM) used, phase assignment and phase pathway

RUN	PRESSURE (GPa)	Δ PRESSURE (GPa)	PTM	PHASE NAME	PHASE PATHWAY
**1**	1.10	–	Ne	**V2**	–
**1**	1.50	0.40	Ne	**V2**	**V2 → V2**
**2**	1.80	–	Ne	**V2**	–
**2**	2.60	0.80	Ne	**V2**	**V2 → V2**
**2**	3.30	0.70	Ne	**V2**	**V2 → V2**
**2**	4.80	1.50	Ne	**V2**	**V2 → V2**
**3**	0.42	–	Ne	**V2**	–
**3**	0.98	0.56	Ne	**V2**	**V2 → V2**
**3**	2.38	1.40	Ne	**V2**	**V2 → V2**
**3**	4.28	1.90	Ne	**V2**	**V2 → V2**
**3**	5.49	1.21	Ne	**V2**	**V2 → V2**
**3**	6.13	0.64	Ne	**V3/V4***	**V2 → V3/V4**
**3**	6.99	0.86	Ne	**V4**	**V3/V4 → V4**
**3**	7.78	0.79	Ne	**V4†**	**V4 → V4†**
**3**	8.50	0.72	Ne	**V4†**	**V4† → V4†**
**3**	9.15	0.65	Ne	**V4†**	**V4 †→ V4†**
**3**	9.88	0.73	Ne	**V4†**	**V4 †→ V4†**
**4**	0.65	–	Ne	**V2**	–
**4**	0.84	0.19	Ne	**V2**	**V2 → V2**
**4**	1.60	0.76	Ne	**V2**	**V2 → V2**
**4**	2.38	0.78	Ne	**V2**	**V2 → V2**
**4**	3.65	1.27	Ne	**V2**	**V2 → V2**
**4**	4.64	0.99	Ne	**V2**	**V2 → V2**
**4**	5.35	0.71	Ne	**V3**	**V2 → V3**
**4**	5.96	0.61	Ne	**V4**	**V3 → V4**
**4**	7.50	1.54	Ne	**V5**	**V4 → V5**
**4**	8.51	1.01	Ne	**V5†**	**V5 → V5†**
**5**	0.59	–	Ne	**V2**	–
**5**	2.54	1.95	Ne	**V2**	**V2 → V2**
**5**	4.58	2.04	Ne	**V2**	**V2 → V2**
**6**	0.59	–	N_2	**V2**	–
**6**	2.18	1.59	N_2	**V2**	**V2 → V2**
**6**	3.49	2.31	N_2	**V2**	**V2 → V2**
**6**	5.95	2.56	N_2	**V3**	**V2 → V3**
**6**	7.18	2.23	N_2	**V5**	**V3 → V5**

**This dataset was a mixed V3/V4 phase which could not be solved.*

†These datasets were single component but only lattice parameters were obtained.

Table S3: Phase angles of all molecules in the asymmetric unit for all phases. E and T denote envelope and twist conformations, respectively. Phase angle for **V1** is calculated from the published structure (REFCODE: VIRAZL)

PRESSURE (GPa)	PHASE	*P* (°)	DESCRIPTOR	SHORTHAND NOTATION
0.00	**V1**	11.1(2)	E C4 -*endo*	E^3
0.00	**V2**	336.0 (4)	E C2 -*exo*	E_2
0.42	**V2**	331.0 (4)	T C1 -*endo*, C2 -*exo*	T_2^1
0.59	**V2**	330.8 (7)	T C1 -*endo*, C2 -*exo*	T_2^1
0.65	**V2**	330.4 (8)	T C1 -*endo*, C2 -*exo*	T_2^1
0.84	**V2**	328.8 (4)	T C1 -*endo*, C2 -*exo*	T_2^1
0.91	**V2**	330.2 (9)	T C1 -*endo*, C2 -*exo*	T_2^1
0.98	**V2**	328.4 (4)	T C1 -*endo*, C2 -*exo*	T_2^1
1.10	**V2**	330.1 (5)	T C1 -*endo*, C2 -*exo*	T_2^1
1.50	**V2**	328.7 (5)	T C1 -*endo*, C2 -*exo*	T_2^1
1.60	**V2**	326.7 (5)	T C1 -*endo*, C2 -*exo*	T_2^1
1.80	**V2**	327.9 (5)	T C1 -*endo*, C2 -*exo*	T_2^1
2.18	**V2**	327.7 (8)	T C1 -*endo*, C2 -*exo*	T_2^1
2.38	**V2**	325.2 (4)	T C1 -*endo*, C2 -*exo*	T_2^1
2.38	**V2**	325.1 (3)	T C1 -*endo*, C2 -*exo*	T_2^1
2.54	**V2**	326.5 (5)	T C1 -*endo*, C2 -*exo*	T_2^1
2.60	**V2**	326.3 (5)	T C1 -*endo*, C2 -*exo*	T_2^1
3.30	**V2**	325.4 (4)	T C1 -*endo*, C2 -*exo*	T_2^1
3.49	**V2**	321.8 (8)	T C1 -*endo*, C2 -*exo*	T_2^1
3.65	**V2**	323.6 (5)	T C1 -*endo*, C2 -*exo*	T_2^1
4.28	**V2**	322.4 (4)	T C1 -*endo*, C2 -*exo*	T_2^1
4.58	**V2**	322.0 (4)	T C1 -*endo*, C2 -*exo*	T_2^1
4.64	**V2**	323.1 (6)	T C1 -*endo*, C2 -*exo*	T_2^1
4.80	**V2**	323.8 (4)	T C1 -*endo*, C2 -*exo*	T_2^1
5.49	**V2**	320.1 (4)	T C1 -*endo*, C2 -*exo*	T_2^1
5.35 (MOL. 1)	**V3**	312.4 (7)	E C11 -*endo*	E^1
5.35 (MOL. 2)	**V3**	320.3 (6)	T C21 -*endo*, C22 -*exo*	T_2^1
5.35 (MOL. 3)	**V3**	6.9 (7)	T C32 -*exo*, C34 -*endo*	T_3^2
5.95 (MOL. 1)	**V3**	310.0 (6)	E C11 -*endo*	E^1
5.95 (MOL. 2)	**V3**	318.7 (6)	T C21 -*endo*, C22 -*exo*	T_2^1
5.95 (MOL. 3)	**V3**	5.9 (7)	T C32 -*exo*, C34 -*endo*	T_3^2
5.96	**V4**	150.4 (6)	T C1 -*exo*, C2 -*endo*	T_1^2
6.99	**V4**	151.0 (7)	T C1 -*exo*, C2 -*endo*	T_1^2
7.18	**V5**	81.7 (7)	E O1 -*endo*	E^0
7.50	**V5**	84.3 (10)	E O1 -*endo*	E^0

Table S4: Intermolecular energies present in the first coordination sphere of the **V4** form of ribavirin at 6.0 GPa. See Figure S12.

Symmetry Transformations	Interaction Label	Interaction Distance (Å)	Coul.	Pol.	Disp.	Rep.	Tot.	Contacts
x+1, *y*, *z*<br>*x*−1, *y*, *z*	*A/A'*	4.947	−118.5	−63.1	−72.8	201.9	−52.6	2xN4H4B···N3 = 1.88 Å, ∠ = 165.32°
x−½, −*y*+½, −*z*+1<br>*x*+½, −*y*+½, −*z*+1	*B/B'*	7.476	−87.5	−46.1	−53.9	142.1	−45.4	2xO2H2···O5 = 1.88 Å, ∠ = 154.86°
x−½, −*y*+³⁄□, −*z*+1<br>*x*+½, −*y*+³⁄□, −*z*+1	*C/C'*	6.197	−57.0	−30.2	−57.9	102.9	−42.2	2xN4H4A···O4 = 2.01 Å, ∠ = 152.54°
−*x*+1, *y*−½, −*z*+³⁄□<br>−*x*+1, *y*+½, −*z*+³⁄□	*D/D'*	8.822	−10.5	−6.5	−17.7	28.5	−6.1	
−*x*+2, *y*−½, −*z*+³⁄□<br>−*x*+2, *y*+½, −*z*+³⁄□	*E/E'*	8.362	−14.3	−10.3	−26.3	43.1	−7.9	
x, *y*+1, *z*<br>*x*, *y*−1, *z*	*F/F'*	6.245	−50.3	−42.6	−37.6	123.3	−7.2	

Coul. = Coulombic, Pol. = Polarisation, Disp. = Dispersion, Rep. = Repulsion, Tot. = Total

Table S5: Intermolecular energies present in the first coordination sphere of the **V4** form of ribavirin at 7.0 GPa.

Symmetry Transformations	Interaction Label	Interaction Distance (Å)	Coul.	Pol.	Disp.	Rep.	Tot.	Contacts
x+1, *y*, *z*<br>*x*−1, *y*, *z*	*A/A'*	4.932	−111.3	−63.7	−73.7	216.0	−32.6	2xN4H4B···N3 = 1.86 Å, ∠ = 165.66°
x−½, −*y*+½, −*z*+1<br>*x*+½, −*y*+½, −*z*+1	*B/B'*	7.574	−82.8	−44.6	−55.1	141.4	−41.1	2xO2H2···O5 = 1.89 Å, ∠ = 153.07°
x−½, −*y*+3/□, −*z*+1<br>*x*+½, −*y*+3/□, −*z*+1	*C/C'*	6.247	−56.4	−30.2	−59.2	104.8	−40.9	2xN4H4A···O4 = 2.02 Å, ∠ = 150.02°
−*x*+1, *y*−½, −*z*+3/□<br>−*x*+1, *y*+½, −*z*+3/□	*D/D'*	8.707	−9.2	−7.7	−19.4	34.2	−2.1	
−*x*+2, *y*−½, −*z*+3/□<br>−*x*+2, *y*+½, −*z*+3/□	*E/E'*	8.362	−21.7	−57.6	−37.4	110.9	−7.9	
x, *y*+1, *z*<br>*x*, *y*−1, *z*	*F/F'*	6.193	−52.2	−29.2	−33.2	107.7	−7.0	

Coul. = Coulombic, Pol. = Polarisation, Disp. = Dispersion, Rep. = Repulsion, Tot. = Total

Table S6: Intermolecular energies present in the first co-ordination sphere of the **V5** form of ribavirin at 7.2 GPa. See Figure S13.

Symmetry Transformations	Interaction Label	Interaction Distance (Å)	Coul.	Pol.	Disp.	Rep.	Tot.	Contacts
x+1, *y*, *z*<br>*x*−1, *y*, *z*	*A/A'*	5.126	−104.3	−58.7	−71.6	191.8	−42.8	2xN4H4B···N3 = 1.94 Å, ∠ = 156.35°
x−½, −*y*+½, −*z*+1<br>*x*+½, −*y*+½, −*z*+1	*B/B'*	7.475	−120.4	−62.1	−64.0	198.2	−48.3	2xO2H2···O5 = 1.79 Å, ∠ = 169.15°
x−½, −*y*+3/□, −*z*+1<br>*x*+½, −*y*+3/□, −*z*+1	*C/C'*	5.888	−33.3	−26.9	−73.3	115.7	−17.8	2xN4H4A···O4 = 2.53 Å, ∠ = 163.56°
−*x*+1, *y*−½, −*z*+3/□<br>−*x*+1, *y*+½, −*z*+3/□	*D/D'*	8.634	−7.5	−14.0	−25.5	49.7	−10.7	
−*x*+2, *y*−½, −*z*+3/□<br>−*x*+2, *y*+½, −*z*+3/□	*E/E'*	8.382	−59.8	−38.2	−31.0	128.9	−0.2	2xO4H4···O3 = 1.86 Å, ∠ = 175.83°<br>2xO3H3···O3 = 2.30 Å, ∠ = 167.93°
x, *y*+1, *z*<br>*x*, *y*−1, *z*	*F/F'*	5.970	−10.5	−7.6	−20.0	27.6	−10.5	

Coul. = Coulombic, Pol. = Polarisation, Disp. = Dispersion, Rep. = Repulsion, Tot. = Total

Table S7: Intermolecular energies present in the first co-ordination sphere of the **V5** form of ribavirin at 7.5 GPa.

Symmetry Transformations	Interaction Label	Interaction Distance (Å)	Coul.	Pol.	Disp.	Rep.	Tot.	Contacts
x+1, *y*, *z*<br>*x*−1, *y*, *z*	*A/A’*	5.091	−112.0	−63.4	−73.7	209.8	−39.3	2xN4H4B···N3 = 1.92 Å, ∠ = 156.70°
x−½, −*y*+½, −*z*+1<br>*x*+½, −*y*+½, −*z*+1	*B/B’*	7.428	−118.5	−70.5	−67.3	215.8	−40.5	2xO2H2···O5 = 1.78 Å, ∠ = 170.39°
x−½, −*y*+³⁄□, −*z*+1<br>*x*+½, −*y*+³⁄□, −*z*+1	*C/C’*	5.837	−34.5	−28.8	−76.5	127.5	−12.3	2xN4H4A···O4 = 2.53 Å, ∠ = 163.71°
−*x*+1, *y*−½, −*z*+³⁄□<br>−*x*+1, *y*+½, −*z*+³⁄□	*D/D’*	8.613	−8.1	−10.2	−25.2	43.4	−0.1	
−*x*+2, *y*−½, −*z*+³⁄□	*E/E’*	8.355	−60.1	−41.5	−33.2	140.1	−5.3	2xO4H4···O3 = 1.85 Å, ∠ = 170.84°<br>2xO3H3···O3 = 2.20 Å, ∠ = 167.84°

$-x+2$, $y+½$,<br>$-z+{}^{3}/$□							
x, $y+1$, z<br>x, $y-1$, z	*F/F’*	5.928	−16.4	−9.6	−22.2	36.7	−11.5

Coul. = Coulombic, Pol. = Polarisation, Disp. = Dispersion, Rep. = Repulsion, Tot. = Total
